# Myeloid-derived immunosuppression of chimeric antigen receptor T cells in the neuronal microenvironment of glioblastoma

**DOI:** 10.1186/s12916-026-04783-2

**Published:** 2026-03-13

**Authors:** Junyi Zhang, Jasmin von Ehr, Thomas Look, Jasim Kada Benotmane, Nicolas Neidert, Jan Kueckelhaus, Tobias Weiss, Dieter Henrik Heiland, Yahaya A. Yabo

**Affiliations:** 1 Department of Neurosurgery, Medical Center - University of Freiburg, Breisacher Straße 64, 79106 Freiburg, Germany; 2 Faculty of Medicine, University of Freiburg, Freiburg, Germany; 3 Microenvironment and Immunology Research Laboratory, Medical Center - University of Freiburg, Freiburg, Germany; 4 Department of Neurology, Clinical Neuroscience Center, University Hospital Zurich and University of Zurich, Zurich, Switzerland; 5 Department of Neurosurgery, University of Hospital Erlangen, Friedrich-Alexander-Universität Erlangen-Nürnberg, Schwabachanlage 6, 91054 Erlangen, Germany; 6 Translational NeuroOncology Research Group, Medical Center - University of Freiburg, Freiburg, Germany; 7 Center for NeuroModulation (NeuroModul), University of Freiburg, Freiburg, Germany; 8 Department of Neurological Surgery, Northwestern University Feinberg School of Medicine, Chicago, USA; 9 German Cancer Consortium (DKTK), Partner Site Freiburg, Freiburg, Germany

**Keywords:** Chimeric antigen receptor, T cell exhaustion, Immunosuppression, Glioblastoma, Brain slices, Transcriptional regulation

## Abstract

**Background:**

Chimeric antigen receptor (CAR)-T cell therapy remains largely ineffective in glioblastoma (GB), where a highly immunosuppressive microenvironment and tumor heterogeneity impair therapeutic durability.

**Methods:**

Using a human neocortical brain slice model that preserves the complex GB microenvironment, we profiled interactions between natural killer group 2D (*NKG2D*) CAR-T cells and tumor ecosystems via PIC-seq, spatial transcriptomics, and gene regulatory network reconstruction.

**Results:**

CAR-T cells showed an early but unsustained tumor-suppressive effect in the slice model. Single-cell profiling revealed that CAR CD8 T cells adopt an effector-skewed activation state accompanied by coordinated upregulation of checkpoint receptors and an exhaustion-associated transcription factor program. These transcriptional changes were linked to ligand-receptor signaling interactions between CAR-T cells and myeloid populations. Tumor-associated macrophages displayed enhanced phagocytic programs and spatially co-localized with mesenchymal-like GB cells within hypoxic regions. Gene regulatory network analysis identified *MAF* and *BACH2* as candidate regulators of CD8 T cell state, with *MAF* enriched in CAR CD8 T cells exhibiting exhaustion-like features, and *BACH2* enriched in Mock CD8 T cells consistent with less differentiated programs. In silico perturbation analyses further suggested a reciprocal effect of *MAF* and *BACH2* on CD8 T cell transcriptional trajectories.

**Conclusions:**

These data map the microenvironmental and transcriptional changes associated with rapid CAR-T cell dysfunction in GB and identify candidate pathways and regulators that may be leveraged to engineer more durable cellular therapies.

**Supplementary Information:**

The online version contains supplementary material available at 10.1186/s12916-026-04783-2.

## Background

Chimeric antigen receptor (CAR)-T cell therapy has shown remarkable success in certain hematologic malignancies; however, this success has not yet been translated to most solid tumors including glioblastoma (GB) [1–6]. GB, characterized by its inherent genetic and cellular heterogeneity as well as its immunosuppressive microenvironment, so far presents an invincible barrier to the efficacy of CAR-T cell therapy [7]. This intratumoral heterogeneity limits the efficacy of CAR-T cells directed against single antigens such as epidermal growth factor receptor deletion mutant variant III (EGFRvIII), human epidermal growth factor receptor 2 (HER2), or interleukin-13 receptor alpha 2 (IL-13Rα2), whose expression is limited to a subset of GB cells even in patients expressing these tumor antigens [5]. This challenge has motivated bivalent/multivalent CAR designs to broaden target coverage [3, 8, 9]. Unlike other single-antigen CARs used against GB, the natural killer group 2D (NKG2D) receptor recognizes multiple stress-induced ligands that are widely expressed by cancer cells, thus providing a wider targeting strategy [10]. NKG2D-based CAR-Ts have been extensively validated in GB and other tumors, supporting their translational potential [11–15].

In contrast to its successes in other malignancies, the limited efficacy of CAR-T cell therapy in GB has raised critical questions regarding its adaptability to the unique challenges posed by the tumor microenvironment (TME) in GB [16]. This immunosuppressive TME dampens the therapeutic potential of CAR-T cells, undermining their ability to mount an effective immune response against the tumor. In GB, T cells transit into a progressive dysfunctional state [17]. This state is characterized by the upregulation of inhibitory receptors, such as PD-1, TIM-3, and LAG-3, which impair the ability of exhausted T cells to recognize and eliminate target cells [18]. Immunotherapeutic approaches, such as immune checkpoint blockade, aimed to revitalize exhausted T cells and restore their anti-tumor activity. However, the persistence of an exhausted phenotype undermines the success of immunotherapies, limiting the durability and strength of the immune response.

In recent studies, a deeper exploration of the GB microenvironment has revealed the importance of T cell-myeloid interaction in driving an immunosuppressive environment. Spatial and single-cell transcriptomic analyses highlight the important role of CD163-positive myeloid cells in promoting this transcriptional transformation in T cells [19]. The mechanism of this phenomenon lies in the HMOX1-IL10 axis and the interplay within mesenchymal tumor regions that influence immune responses in gliomas [18]. CAR-T cell therapy has shown notable efficacy in cell lines, murine and patient-derived xenograft (PDX) models, with no apparent signs of T cell exhaustion [20–23]. However, in human clinical reports, T cell exhaustion has emerged as a significant impediment to the effectiveness of CAR-T therapy. Thus, a comprehensive and detailed exploration of the regulatory mechanisms governing CAR-T cell functionality within the intricate GB ecosystem is necessary in a model closer to human GB. Here, we compared CAR-T efficacy with normal T cells (which lack the CAR construct) in vitro using a novel human neocortical slice model, in which we used the access cortex from neurosurgery and implanted mesenchymal primary-derived tumor cells in the brain slices [24]. Additionally, CAR and control T cells were added to the tumor-implanted brain slices and profiled using PIC-seq [25].

We hypothesize that the human neural environment, particularly in conjunction with mesenchymal tumors, will recapitulate the human GB TME and may manifest distinct responses compared to murine models. Further, we hypothesize that these distinct responses mask the effects of immune suppression observed in PDX models which in turn necessitates the need for a more comprehensive understanding of the interplay between CAR-T cell therapy, neural environments, and tumor subtypes in patients [26].

Our integrative analysis of CAR-T and Mock-treated brain slices revealed that the brain slice model recapitulates the GB tumor microenvironment. We revealed that, upon interaction with GB cells, CAR-T cells exert an initial antitumor effect followed by a decline in effectivity. We identified a subpopulation of tumor-associated macrophages (TAMs) with enhanced phagocytic and scavenger activity spatially located in proximity to mesenchymal-like GB cells within hypoxic tumor niches potentially contributing to CD8 T cell dysfunction. Both myeloid-tumor and myeloid-T cell interactions collectively promote an immunosuppressive microenvironment in GB that drives transcriptional shift towards T cell exhaustion. Gene regulatory network analysis identified *MAF* and *BACH2* as key transcriptional regulators of CAR-T cell function. Our findings provide insights into the molecular mechanisms governing the functionality of CAR-T cells and highlight *BACH2* and *MAF* as potential targets for optimizing and enhancing the efficacy of CAR-T cell therapy.

## Methods

### Human organotypic slice culture

We used a human neocortical slice model recently described in detail [24]. In the case of tumors deeply localized without infiltrating the cortex, we used cortical regions that were removed to access the tumors, followed by an intraoperative evaluation using Stimulated Raman Histology to confirm the non-tumor infiltration status. The cortical tissue was collected and transported to the laboratory in a pre-cooled preparation medium within 10 min of the resection procedure. In brief, the tissue block was processed on a pre-cooled platform. All visibly cauterized and damaged parts were dissected away before trimming the tissue into smaller cubes. These cortex cubes were fixed on a magnetic platform and re-sliced into 300 µm thick slices using a Leica VT1200 semi-automatic vibratome (14,912,000,001, Leica, Germany) and cultured for this study. Growth medium was used as slice culture medium and was refreshed every 24 h throughout the culture period. Brain slices were allowed to recover for the initial 24 h postcollection.

### Tumor inoculation

To establish the GB brain slice model, 1 μL of ZsGreen-tagged BTSC233 GB cells (20,000 cells/μL) was injected into each brain slice. Tumor growth was monitored every 24 h using an EVOS M7000 fluorescent microscope paired with an on-stage incubation system. On day 3 post-tumor injection (DPI3), CAR-T cell, Mock T-cell, or control treatment was applied by injecting Cell Trace Far Red (CTFR)-tagged single T-cell suspensions. A total of 2 million cells in 4 μL were injected per brain slice. Tumor growth was assessed at DPI3 to confirm model reliability. Slices that did not exhibit established tumor patterns at DPI3 were excluded from further experiments; no such exclusions occurred in this study.

#### Generation of CAR-T cells

Human T cells were isolated from Peripheral Blood Mononuclear Cells (PBMCs) using the EasySep™ Release Human CD3 Positive Selection Kit (Stemcell Technologies, #17,751). Enriched T cells were activated with Dynabeads™ Human T-Activator CD3/CD28 for T Cell Expansion and Activation (Thermo Fisher, #11131D) for 3 days and kept at a density of 1 × 10^6^ cells/ml. The medium was supplemented with 100 U/ml IL-2 throughout the culture. T cells were expanded for 11 days and then electrophoresed with human NKG2D-CAR messenger ribonucleic acid (mRNA) using a NEON transfection system (Invitrogen). In vitro transcription and electroporation of human NKG2D-CAR mRNA have recently been described [14]. For CAR-T cell generation, 10 µg mRNA was used per 100 µl electroporation reaction.

### Live imaging and tumor growth monitoring

Imaging was performed using an upright two-photon microscope Olympus FV1000 using an excitation wavelength of 870–890 nm with an emission filter of 515–560 nm to image the green fluorescent protein (GFP) signal. Optical magnification was set to 20 × . Images were acquired with 1- to 2-μm *z*-axis increments and 800 × 800-pixel resolution. Simple neurite tracer (SNT) plugin^42^ implemented in ImageJ was used to visualize cell–cell connections. We transformed the traces into a 3D matrix and visualized the network pattern in plotly (R). Temporally resolved live tissue imaging was performed using an EVOS M7000 microscope (AMF7000, Thermo Fisher Scientific, USA) with an on-stage incubator (AMC1000, Thermo Fisher Scientific, USA) coupled with an automated temperature, humidity, O_2_, and CO_2_ controlling system. Image acquisition interval was 24 h. GFP and Cy5 filter cubes were used respectively for acquiring images for tumor cells or CAR-T/Mock cells under an Olympus UPLXAPO 4X objective (#14–905, EVIDENT Olympus, Japan). Tumor growth was quantified as below:

$$Tumor\;density\;index=Tumor\;growth\;area\;\times\;Mean\;fluorescent\;intensity\;of\;the\;tumor\;growth\;area$$

A *t*-test was used to compare the daily tumor density increase between CAR-T-cell treated and Mock T-cell treated groups. For day (*n*) posttreatment:

$$Daily\ tumor\ density\ increase = [Tumor\ density\ index\ (n) - Tumor\ density\ index\ (n-1)] / Tumor\ density\ index\ (n-1)\ \ast\ 100\%$$

### Tissue dissociation

For tissue dissociation and preparation of single-cell suspensions, samples were processed using the Neural Tissue Dissociation Kit (Miltenyi Biotec) according to the manufacturer’s protocol with slight modifications. Briefly, enzyme mix 1 was prepared by combining 200 μL of Enzyme T with 1750 μ of Buffer X per 500 mg of tissue, and enzyme mix 2 was prepared by mixing 10 μL of Enzyme A with 20 μL of Buffer Y per 500 mg of tissue. Tissue samples were transferred into C-tubes, supplemented with enzyme mix 1, and incubated at 37 °C for 5 min. Mechanical dissociation was then performed within the C-tube for 2 min at 37 °C under slow, continuous rotation. Subsequently, enzyme mix 2 was added and the sample was incubated for an additional 5 min at 37 °C, followed by a second round of mechanical dissociation under the same conditions. The dissociated suspension was centrifuged at 350 g for 1 min at 4 °C, the supernatant was removed, and the cell pellet was resuspended in 9 mL of growth medium. The suspension was then filtered through a 100-μm cell strainer (pre-moistened with 1 mL growth medium) placed atop a 50-mL falcon tube. The filtrate was collected and transferred to a 15-mL falcon tube for downstream applications.

### Fluorescence-activated cell sorting

Fluorescence-activated cell sorting (FACS) for brain slice culture was recently described by us [19, 24]. Briefly, single-cell suspensions were centrifuged at 300 g for 10 min at 4 °C. Following centrifugation, the supernatant was aspirated, and the pellet was gently resuspended. The resuspended cells were transferred to FACS tubes containing FACS buffer and centrifuged again at 300 g for 5 min at 4 °C. Washed samples were resuspended in 450 μL DAPI-containing FACS incubation buffer (DFIB) and incubated on ice, protected from light. A total of 70,000 DAPI-negative, ZsGreen-positive cells were sorted by collecting two sets of 35,000 cells into pre-coated Eppendorf tubes for downstream analysis. The pellets were then gated and separated into different compartments based on the fluorescence of either Alexa488 or Alexa647 or both. scRNA-seq was performed on the different compartments of interest.

### Single-cell RNA sequencing

PIC-seq, a modified scRNA-seq, was employed in this study using 10 × Genomics Chromium Next GEM Single Cell 3′ Reagent Kits User Guide (v3.1 Chemistry). Amplified cDNA and constructed single-cell libraries were evaluated using Fragment Analyzer 5200 (M5310AA, Agilent, USA) and Qubit™ 4 Fluorometer (Q33238, Thermo Fisher Scientific, USA) for quality assessment of base pair length and concentration. The constructed libraries were indexed, denatured, and pooled based on Illumina NextSeq 500 and 550 System Denature and Dilute Libraries Guide. NextSeq High Output kit v2.5 (20,024,906, Illumina, USA) flowcell chip (75 cycles) was used. Sequencing cycles were set as follows: read1 – i7 – i5 – read2: 28 – 8 – 0 – 56.

### scRNA-seq data analyses

Gene expression matrix from the CellRanger pipeline was analyzed using the Seurat package in R. Low-quality cells (cells expressing less than 200 genes, and genes expressed in less than 5 cells plus cells with mitochondrial reads greater than 10%) were filtered out. Data were log-transformed to mitigate technical noise and gene expression variability. Cells were clustered using the FindNeighbors and FindClusters functions in Seurat. Principal Component Analysis (PCA) was applied to reduce the dimensionality of the data, and cell clusters were identified using shared nearest neighbor (SNN) modularity optimization. Clusters were visualized using Uniform Manifold Approximation and Projection (UMAP) to group cells with shared gene expression profiles. To account for batch effects resulting from data generated from different conditions, the Mutual Nearest Neighbors (MNN) correction method was applied. This method aligns the data across batches to enable the comparison of gene expression profiles. MNN-corrected data were used for downstream analysis. Differentially expressed genes (DEGs) were determined using the Wilcoxon rank-sum test to identify genes associated with specific cell types and experimental conditions.

PHATE (Potential of Heat-diffusion for Affinity-based Transition Embedding) [27] dimensionality reduction was performed to visualize the continuous transcriptional landscape of CD4 and CD8 T-cell populations. The normalized expression matrix from the Seurat object was transposed to ensure cells were represented as rows and used as input for the phate()function from the phateR package. The resulting two-dimensional PHATE embeddings (PHATE1 and PHATE2) were added to the Seurat object metadata and visualized using FeatureScatter to examine sample distribution across conditions and groups. To explore gene-specific expression patterns along the inferred cellular manifold, expression levels of selected genes (*MAF* and BACH2) were mapped onto the PHATE embedding using ggplot2.

Pseudotime trajectory analysis was performed using the slingshot R package to infer potential lineage relationships and dynamic transcriptional changes within CD4 and CD8 T-cell populations [28]. UMAP embeddings and corresponding cluster identities from the Seurat object were used as input to the slingshot() function, which fits smooth curves through the reduced-dimensional space to model continuous developmental trajectories. Pseudotime values were extracted from the inferred lineages and incorporated into the Seurat metadata for downstream visualization. Cells were plotted along the UMAP embedding and colored by pseudotime to depict progression along the inferred trajectory. To explore gene dynamics across pseudotime, the expression patterns of key transcriptional regulators (*BACH2* and *MAF*) were smoothed using LOESS regression with ggplot2, enabling visualization of gradual changes in gene expression along the continuum of T cell differentiation or activation states.

Copy number variation (CNV) inference was performed using the inferCNV R package to identify large-scale chromosomal alterations at the single-cell level. A Seurat object containing raw count data was used as input, with T cells, oligodendrocytes, and myeloid cells designated as the reference population for CNV normalization. The CreateInfercnvObject()function was used to construct the inferCNV object, integrating the raw counts matrix, gene positional information (based on hg38 gencode v27), and cell-type annotations. The run() function was executed with a cutoff of 0.1 to filter lowly expressed genes, and denoising and hidden Markov model (HMM) options were enabled to enhance signal detection and identify discrete CNV states. Hierarchical clustering was applied to group cells by CNV profiles, and the resulting CNV heatmaps were visualized using plot_cnv(), enabling the detection of chromosomal gains and losses across different cell types relative to the reference.

### Doublet scoring and removal

Doublets were identified and removed using the DoubletFinder R package [29]. Based on our FACS analyses, we generated separate scRNA-seq libraries for Cy5⁺, ZsGreen⁺, and Cy5⁺ZsGreen⁺ (double-positive) populations. The latter served as an internal control for true biological doublets. To ensure accurate doublet detection, DoubletFinder was applied independently to each sample. Optimal parameters were determined using the paramSweep_v3, summarizeSweep, and find.pK functions, selecting the local maximum of the BCmvn distribution as the optimal pK. The expected doublet rate (nExp) was estimated empirically and adjusted for homotypic doublets using modelHomotypic. DoubletFinder was then run with optimized parameters (pN = 0.25, pK = sample-specific value, nExp = adjusted expected rate). Cells classified as doublets within the Cy5⁺ and ZsGreen⁺ samples were removed as technical doublets, while Cy5⁺ZsGreen⁺ double-positive doublets were retained as biologically relevant populations.

### Cell type annotation

We first used the Seurat function FindAllMarkers to identify differentially expressed genes for each of the 4 major clusters. The major cell types (tumor, T cells, myeloid, oligodendrocytes) were identified by the top marker genes from the FindAllMarkers result. To further identify the subpopulations in the major cell types, we used Azimuth to map our scRNA-seq data to a known glioblastoma reference dataset called GBmap using the annotation_level_4 cell types. To compare cell-type classifications between the major cell types and GBmap cell type, we generated a contingency matrix summarizing the overlap between annotated clusters from each dataset. Cluster identities were extracted as categorical variables and cross-tabulated to quantify the number of shared cells per cluster pair. The resulting matrix was organized by major cell type order and visualized using a Sankey diagram implemented in the *flipPlots* R package. This visualization illustrated the correspondence and relative proportions of cells across the two annotation systems, enabling assessment of concordance between the major cell types and the reference clusters.

### Gene ontology enrichment

Gene Ontology (GO) enrichment analysis was performed to identify biological processes significantly associated with differentially expressed genes from the CAR-T and Mock conditions. Enrichment was assessed for the Biological Process (BP) ontology using Benjamini–Hochberg correction for multiple testing, with adjusted *p*- and *q*-value cutoffs of < 0.05. The top enriched GO terms were visualized using bar plots and dot plots to display the most significantly overrepresented biological processes in each condition.

### Module score calculation

Exhaustion module scores were calculated independently for a list of 20 curated CD8 T cells exhaustion markers, exhaustion-related transcription factors, CD8 T cells effector and stem-like genes as well as GSE9650 CD8 T cell signature [30]. Scores were computed for individual cells using the Seurat AddModuleScore function, which calculates the average expression of genes within each signature relative to a control gene set matched for expression levels. This approach accounts for technical variation and provides a single-cell level quantification of exhaustion program activity. The list of genes for each of the modules is in Additional file 2: Table 4. Exhaustion scores were compared between CAR-T and Mock groups using the Wilcoxon rank-sum test (Mann–Whitney *U* test), a non-parametric test appropriate for single-cell expression data that does not assume normal distribution. Effect sizes were quantified using Cohen’s *d*, calculated as the difference in group means divided by the pooled standard deviation.

$$d = \frac{\bar{x}_{CAR} - \bar{x}_{Mock}}{S_{pooled}}$$

### Cell-to-cell communications

Cell-to-cell interaction inference was done using the CellChat package (v1.6.0). In this analysis, the 'CellChatDB.human' database was used as a reference source for understanding cell-to-cell communication pathways between distinct cell types in various experimental conditions (CAR-T vs Mock). We first identified activated signaling pathways in each specific experimental condition. Subsequently, the 2 conditions were merged to compare the signaling pathways between them as described in the CellChat tutorial on comparing “multiple datasets with different cell type composition.” We identified dysfunctional signaling by comparing the communication probabilities and differential expression analyses between CAR-T and Mock groups.

### Gene regulatory network construction

Before the construction of the gene regulatory networks (GRNs), only CD8 cells were extracted from the Seurat object and converted to H5ad format. CellOracle (version 0.18.0) was used to infer cell-specific gene regulatory networks and predict the activity of transcription factors within individual cells using scRNA-seq. TFs controlling the gene regulation in both groups were identified based on the calculated centrality scores. The selected TFs were knocked out to simulate cell state transitions in each group. Pseudotime trajectory was used to identify the normal developmental path of the cells. The gene regulatory interactions were filtered to allow only connections where the *JUN* or *FOS* was either a source or target gene.

### Spatial transcriptomics data analysis

Spatial transcriptomics data from multiple samples were processed and analyzed to infer the adjacency and relationship between different cell types. For each dataset, a correlation matrix was computed to quantify the adjacency between the selected cell types. The individual correlation matrices were combined into a single matrix representing the mean correlation values. The resulting mean correlation matrix was filtered to retain only significant correlations, defined as those with a value greater than 0.4. The processed data served as the basis for constructing a network graph, where nodes represent cell types, and edges indicate significant correlations between them. The graph was constructed using the `igraph` package, with edges directed and properties such as magnitude and color derived from the correlation data. Visualization of the network graph was performed using “ggraph.”

## Results

### CAR-T cells display an initial anti-tumor effect on glioblastoma cells in a complex human-like microenvironment

**Fig. 1:**
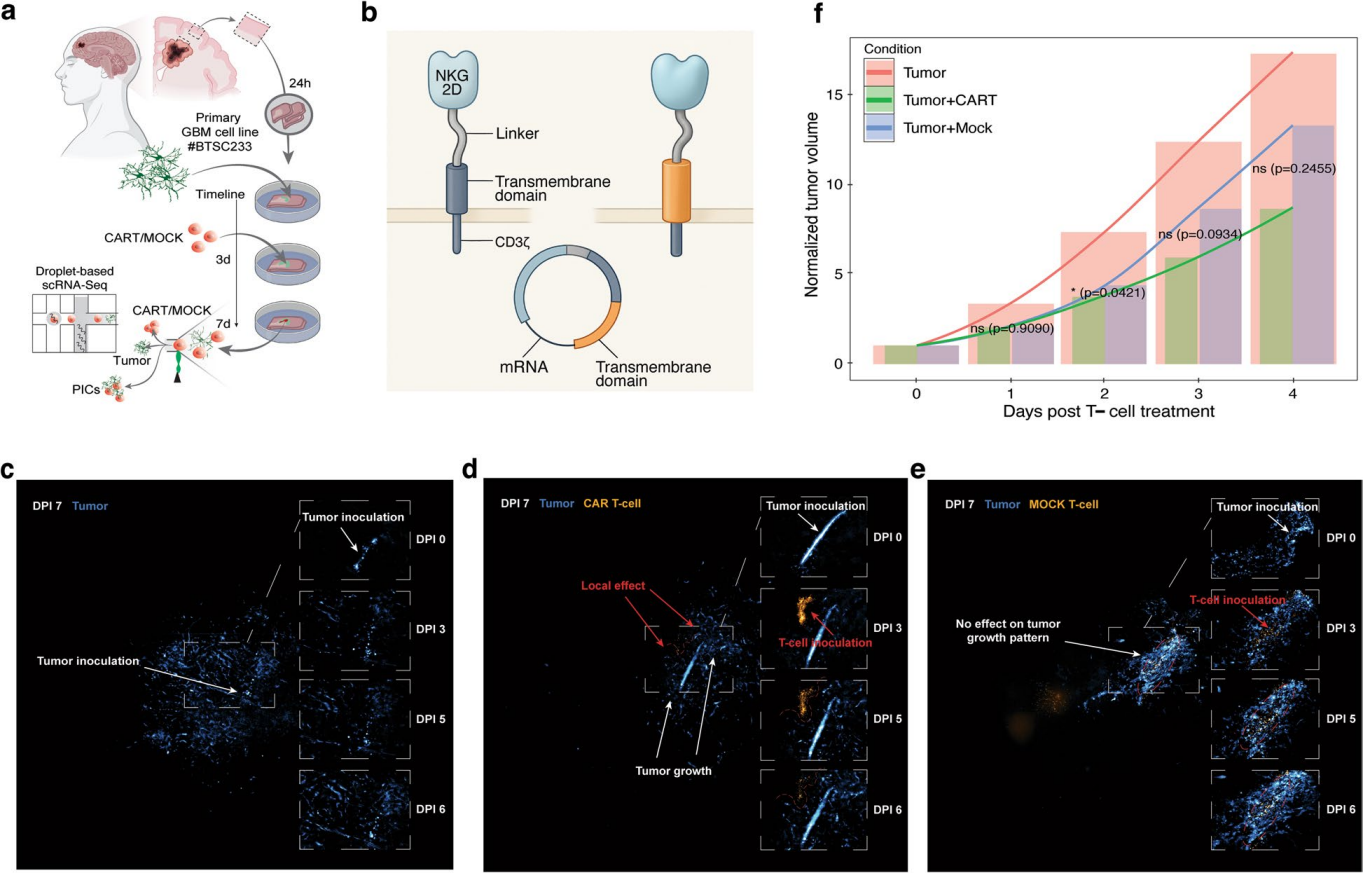
Impact of CAR-T cell treatment on tumor growth in glioblastoma inoculated brain slices. **a** Illustration of the experimental workflow showing brain slice culture, CAR-T cell treatment, and droplet-based scRNA-seq. **b** Illustration of the NKG2D CART construct. **c, d**, and** e** Representative tumor growth pattern respectively in control, CAR T cell, and mock conditions (DPI = day post-inoculation, scale bar = 1 mm). **f** Quantification of tumor growth under CAR-T/mock treatment, as well as the respective control condition. (Tumor density index = Area x mean_intensity). Error bar = mean ± SEM. PICs = physically interacting cells

To understand GB-CAR T cells interaction in a human-like model with intact neuro-glial microenvironment, we employed a GB-injected brain slice culture model treated with CAR-T cells, coupled with high-throughput transcriptomic analysis. Slices of 300 μm thickness were prepared and cultured in a growth medium. The medium was refreshed every 24 h throughout the culture period. Brain slices were allowed to recover for the initial 24 h post-collection before tumor cells injection [19, 24] (Fig. 1a). We inoculated the brain slices with the heterogeneous primary cell line (#BTSC233), which has already demonstrated a high level of plasticity in previous studies [19]. To ensure the consistency of the model, we generated and inoculated brain slices across three different patients with the same cell line (#BTSC233). Tumor cell proliferation and growth were monitored microscopically. After 3 days of tumor growth, the brain slices were treated with either T cells with a CAR construct (CAR-T) (Fig. 1b) or control PBMC-derived T cells without CAR construct (Mock). Slices injected with only GB cells without T cell treatment showed a progressive growth and infiltration of the GB cells in the slices (Fig. 1c). We observed a decline in GB cells growth based on the area covered by the tumor cells in the slices in both the CAR-T and Mock groups. In both the Mock and CAR-T treated slices, a tumor-suppressing local effect during the early phase following CAR-T cell inoculation was observed (Fig. 1d, e). The tumor suppressing effect observed in the Mock condition may be explained by a non-antigen restricted cytotoxic effect from the peripheral blood–derived T cells (without a CAR). This is consistent with early activation-driven killing due to the bystander effect seen in conventional T cells [31]. This effect was short-lived, and tumors subsequently regrew as Mock T cell function waned. Quantification of the tumor area revealed significant decrease (*p* = 0.0421) in tumor growth in CAR-T treated brain slices in the initial phase post-treatment, in comparison to Mock treated and untreated conditions. However, the differences between CAR-T and Mock groups are not statistically significant at days 3 and 4 (Fig. 1f). These observations indicate a potential therapeutic local effect exerted by the CAR-Ts indicating a trend toward greater tumor control by the CAR-Ts. However, the subsequent decline in T cell levels with an increase in GB cells growth following this initial phase of CAR-T hyperactivity suggests that the CAR-T cells may have lost their anti-tumor effect potentially due to exhaustion.

### Single-cell analyses of glioblastoma cells and the tumor microenvironment in neocortical slices

**Fig. 2:**
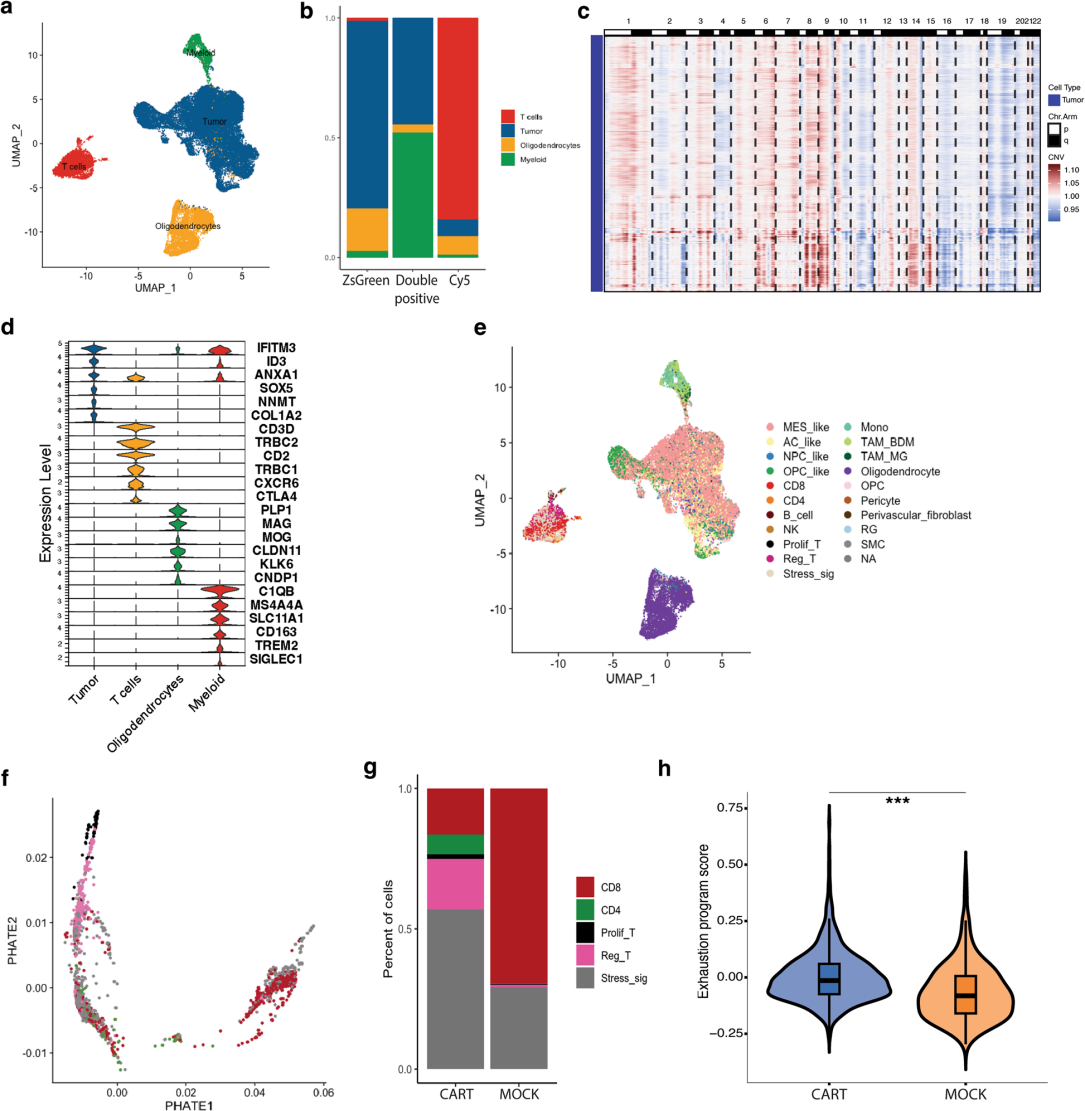
scRNA-seq analysis of brain slices treated with CAR and normal T cells. **a** UMAP representation showing major cell types identified by marker expression. **b** Barplot showing the proportions of cell types in each channel. **c** Heatmap of inferred copy number alterations, on the *Y*-axis are cells and on the *X*-axis are the chromosomes. **d** Violin plot of key marker genes for myeloid, T cells, oligodendrocytes, and tumor cells. **e** UMAP showing cell types mapped to GBmap reference dataset. **f** PHATE map of T cell subpopulations showing the identified populations ordered along a transcriptional landscape. Cells are colored by annotated subpopulations in Fig. 2 g. **g** Stacked bar chart of T cell subsets showing the different T cell subpopulation and their proportions in CAR T cell and mock T cell groups. **h** Violin plot of exhaustion scores calculated from a 20-gene signature of inhibitory receptors and transcriptional regulators. Violin shapes show score distribution; embedded box plots show median and quartiles. Statistical significance was assessed using the Wilcoxon rank-sum test. ***p < 0.001, **p < 0.01, *p < 0.05, ns not significant. MES mesenchymal-like, AC astrocytic-like, NPC neural progenitor cell-like, OPC oligodendrocyte progenitor cell-like, NK = natural killer cell, Prolif_T proliferating T cells, Reg_T regulatory T cells, Stress_sig T cells with stress signature, Mono = monocyte, TAM_BDM bone marrow-derived tumor-associated macrophages, TAM_MG microglia derived tumor-associated macrophages, RG radial glia, SMC = smooth muscle cells

To identify the cellular diversity within the brain slices injected with CAR-T cells and normal T cells, at day 7 post-CAR-T injection, the slices were dissociated and FAC (Fluorescence-activated cell) sorted into 3 major channels, T cells (Cy5^+^), tumor cells (ZsGreen^+^), and double-positive cells considered as physical interacting cells (double positive = Cy5^+^ZsGreen^+^) [25] (Fig. 1a). We performed droplet-based physically interacting cells sequencing (PIC-seq) that combines cell sorting combined with scRNA-seq on the different groups. The resulting scRNA-seq data from different samples (Additional file 1: Fig. S1a) were merged and horizontally integrated using the mutual nearest neighbor algorithm (MNN) with the top 2000 most variable features as anchors. After quality control, a total of 35,085 cells remained (Fig. 2a). We identified malignant cells through inferred copy-number alterations (Fig. 2c, Additional file 1: Fig. 1b). Next, we identified the major cell types by the top markers expressed by each cluster (Fig. 2d) from a list of top differentially expressed genes per cluster (Additional file 2: Table 1). We then mapped our data to the reference GBmap dataset to infer cell types using Azimuth [32] (Additional file 1: Fig. 1c). Our initial cell type identification corresponds to the major GBmap cell types [33] (Fig. 2e, Additional file 1: Fig. 1d).

To characterize the transcriptional diversity within the T cell compartment, we performed subclustering of T cells and visualized their transcriptional relationships using PHATE dimensionality reduction [27]. The resulting PHATE map revealed a continuous developmental trajectory, with proliferating and regulatory T cells placed at one end and CD8 T cells at the opposite end. CD4 T cells were mostly located in the middle of the trajectory, suggesting transitional transcriptional states between regulatory and cytotoxic lineages. Although not a direct measure of lineage progression, the PHATE manifold captures a continuum of transcriptional states that likely represents functional differentiation among T cell subsets. Notably, T cells expressing stress-associated gene signatures were broadly distributed along the continuum, indicating that stress responses occur across multiple transcriptional states rather than being confined to a specific subset (Fig. 2f). The T cell subsets consist of clusters enriched for *CD8A*, *CD4*, *TOP2A*, *CTLA4*, and *JUN*, representing CD8 T cells, CD4 T cells, proliferating T cells, regulatory T cells, and T cells with stress signatures respectively (Fig. 2f, Additional file 1: Fig. 1e, Additional file 2: Table 2). A comparison of T cell subpopulation between the CAR-T and Mock populations revealed the presence of the identified T cell subtypes in all groups. CD4 T cells were however absent in the Mock group (Fig. 2g). Since CD8 T cells are known to exhibit rapid exhaustion after an initial antitumor effect in comparison to GB [34] and to allow proper comparison between the CAR-T cell and Mock T cell groups, we focused on CD8 T cells in our downstream analysis.

To understand the phenotypic diversity of the CD8 cells in the different groups, we first identified the differentially expressed genes (DEGs) between CAR-T and Mock CD8 T cells (Additional file 2: Table 3). Gene ontology analysis of these DEGs (Supplementary Fig. 1f) revealed an enrichment of translational, nucleotide, and energy-metabolic processes that is consistent with ongoing T cell activation under chronic stimulation that is progressing toward an exhausted state. This result aligns with reports showing that chronically stimulated CD8 T cells can show an enrichment of anabolic and oxidative phosphorylation [35–37]. In contrast, the enrichment of lymphocyte and T-cell differentiation as well as G1/S transition of mitotic cell cycle in the Mock T cells indicates a homeostatic T cell proliferation without hallmarks of chronic stimulation–driven dysfunction [38]. Taken together, the observed anabolic metabolism in the CAR-T group strongly suggests chronic activation under immunoregulatory, impaired ADP-coupled oxidative phosphorylation contributing to an exhaustion trajectory [35, 38]. We then checked the expression of different T cell markers (Naïve, activated, and exhausted) in the CD8 subpopulation in the CAR-T and the Mock T cell groups to understand the cause of this aberrant T cell metabolism in the CAR-T group. Naive T cell markers (*SELL*, *CCR7*, *CD8A*) and activation markers (*GZMB*, *NKG7, GZMA, EOMES*) were highly expressed in Mock CD8 T cells in comparison to the CAR-T group, while T cell exhaustion markers (*LAG3, CTLA4, TOX2, PDCD1*) were highly expressed in the CAR-T group (Additional file 1: Fig. 2a). However, some canonical T cell exhaustion markers (*TOX* and *NR4A2*) were not differentially expressed compared to the Mock group, implying a non-uniform expression of exhaustion markers in the CAR-T population. CAR-T cells also show elevated expression of *ZEB2* and *PRDM1(BLIMP1)* compared with the Mock group, consistent with enhanced effector differentiation, a state frequently associated with CAR-T cell dysfunction [39–41]. To better define the T cell states, we quantified T cell exhaustion using program-level module scoring rather than individual markers. A 20-gene exhaustion program score was significantly higher in CAR-T cells than Mock (Cohen’s *d* = 0.57, *p* = 1.44 × 10^−15^) (Fig. 2h), indicating enrichment of an exhaustion-associated transcriptional program in CAR-T cells. Submodule analysis showed increased transcription factor-associated exhaustion in CAR-T cells (*p* = 4.56 × 10^−46^), whereas both the stem-like (*p* = 1.27 × 10^−05^) and effector (*p* = 4.68 × 10^−46^) modules were significantly higher in Mock (Additional file 1: Fig. 2b). Consistently, an independent MSigDB exhaustion reference signature [30, 42] was also enriched in CAR-T cells (*p* = 2.87 × 10^−05^) (Additional file 1: Fig. 2c, Additional file 2: Tables 4 and 5). Taken together, the rapid decline in antitumor effect observed in the brain slices treated with CAR-T cells coincides with transcriptional features of dysfunction and terminal differentiation along with exhaustion-like features in the CAR CD8 T cell subpopulation, consistent with previous reports [34].

### Cell–cell signaling reveals CAR CD8 T cells dysfunction upon interaction with the glioblastoma microenvironment

**Fig. 3:**
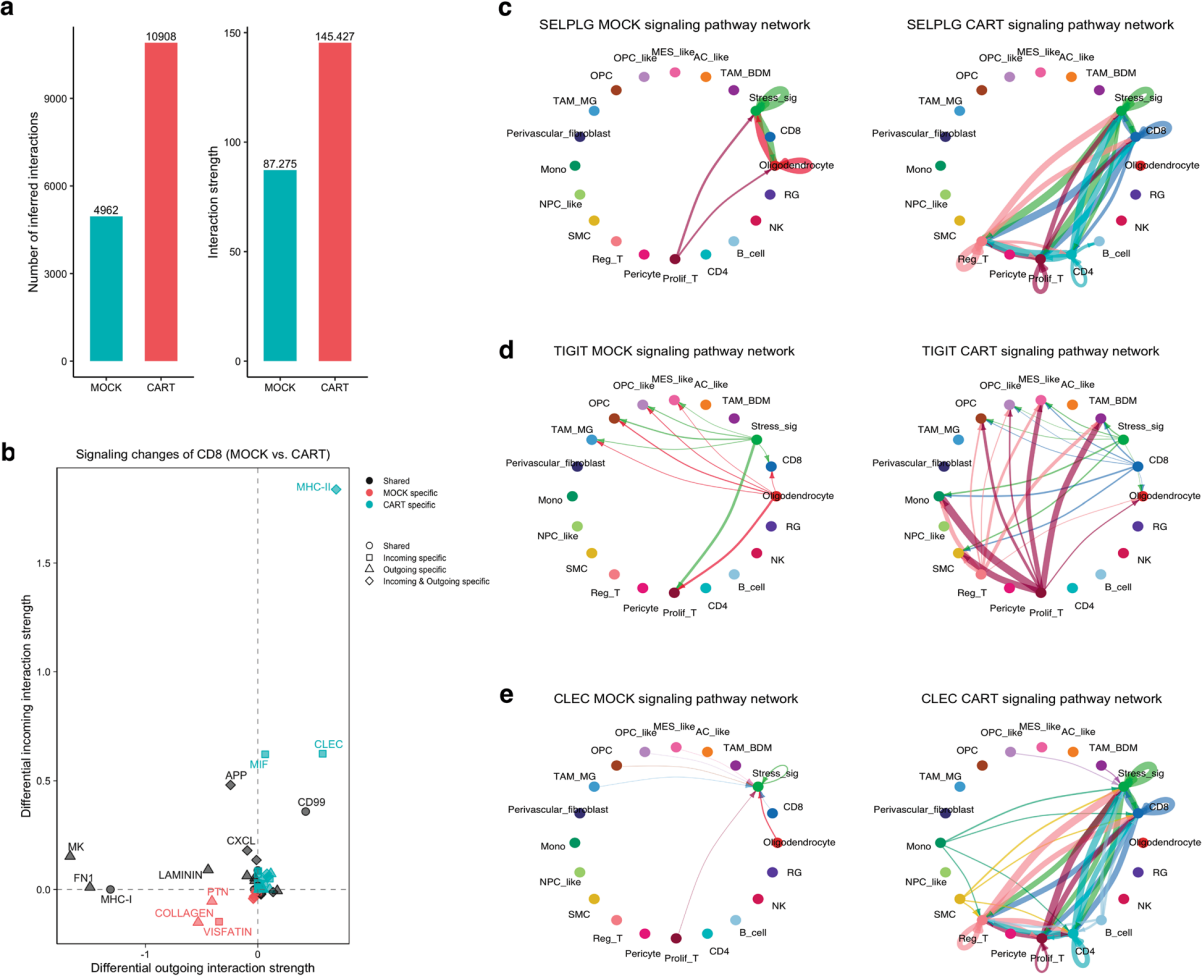
Cell–cell communication between CD8 T cells and cells within the GB microenvironment. **a** Total number of interactions and the strength of the interactions in mock and CAR-T cell groups. **b** Signaling changes of CD8 cells in mock compared to the CAR-T cell group. **c, d**, and** e** Circle plots showing the signaling directions of key signaling pathways upregulated in the CAR-T cell group, SELPLG, TIGIT and CLEC respectively

To understand the role of the TME in CAR-T cells function or dysfunction during therapy in GB, we used CellChatDB [43] to identify the communication patterns between all cells within the GB TME in both CAR-T and Mock-treated GB cells. First, we identified all the signaling pathways activated in CAR-T and Mock groups. Our analysis revealed a significant difference in ligand-receptor interactions and the strength of the interactions between the CAR-T and the Mock group (Fig. 3a). The CAR-T group also had a higher activation of signaling pathways indicating enhanced cellular communication between CAR-T cells and other cell types within the TME (Additional file 1: Fig. 3b). Thus, we hypothesized that this increased cellular signaling within the TME of CART treated GB cells contributes to T cell dysfunction and treatment failure. Interestingly, among the pathways enhanced in the CAR-T group when compared to the Mock group are TIGIT, PD-L1, SELPLG, NOTCH, that are strongly linked to T cell exhaustion (Additional file 1: Fig. 3a). Given our initial observation that CD8 T cells are the most abundant T cell subpopulation and are present in all the groups in our dataset, we focused our analysis on the signaling between CD8 T cells and other cells within the GB TME. We confirmed the upregulation of T cell exhaustion pathways in the CAR-T group compared to the Mock group by the significant activation of SELPLG and TIGIT pathways that indicate T cell exhaustion (Fig. 3c and d). Additionally, we found an upregulation of signaling pathways (CLEC, MHC-II, and MIF) specific to CD8 CAR-T cells (Fig. 3b). Notably, the CLEC pathway involving the ligands *CLEC2B*, *CLEC3C*, and *CLEC3D* expressed by several immune cells interact with the CD161 (*KLRB1*) receptor expressed by exhausted CD8 T cells (Fig. 3e). Interestingly, regulatory T cells, proliferating T cells, and CD4 T cells all interact with CD8 T cells via the CLEC-KLRB1 pathway in the CAR-T group. Surprisingly, we observed an upregulation of MHC-II signaling (Additional file 1: Fig. 3c) in the CAR-T group involving the CD8 cells, and a downregulation of the canonical MHC-I pathway (Additional file 1: Fig. 3d) further indicating the dysfunctional signaling in CD8 CAR-T cells. It is worth noting that, while CAR-T cells in our dataset show enrichment of pathways related to T cell exhaustion, they also show enrichment of pathways linked to T cell activation including IL2, CD80, CD86, CD22, and CD40. These findings suggest that the CAR-T cells may exhibit early exhaustion-like or activation-dysregulation signatures, rather than fully established exhaustion. Taken together, our results reflect transcriptional and functional states consistent with early activation-induced dysfunction, rather than terminal exhaustion.

### Myeloid doublets exhibit enhanced phagocytic activity in proximity to glioblastoma cells

**Fig. 4:**
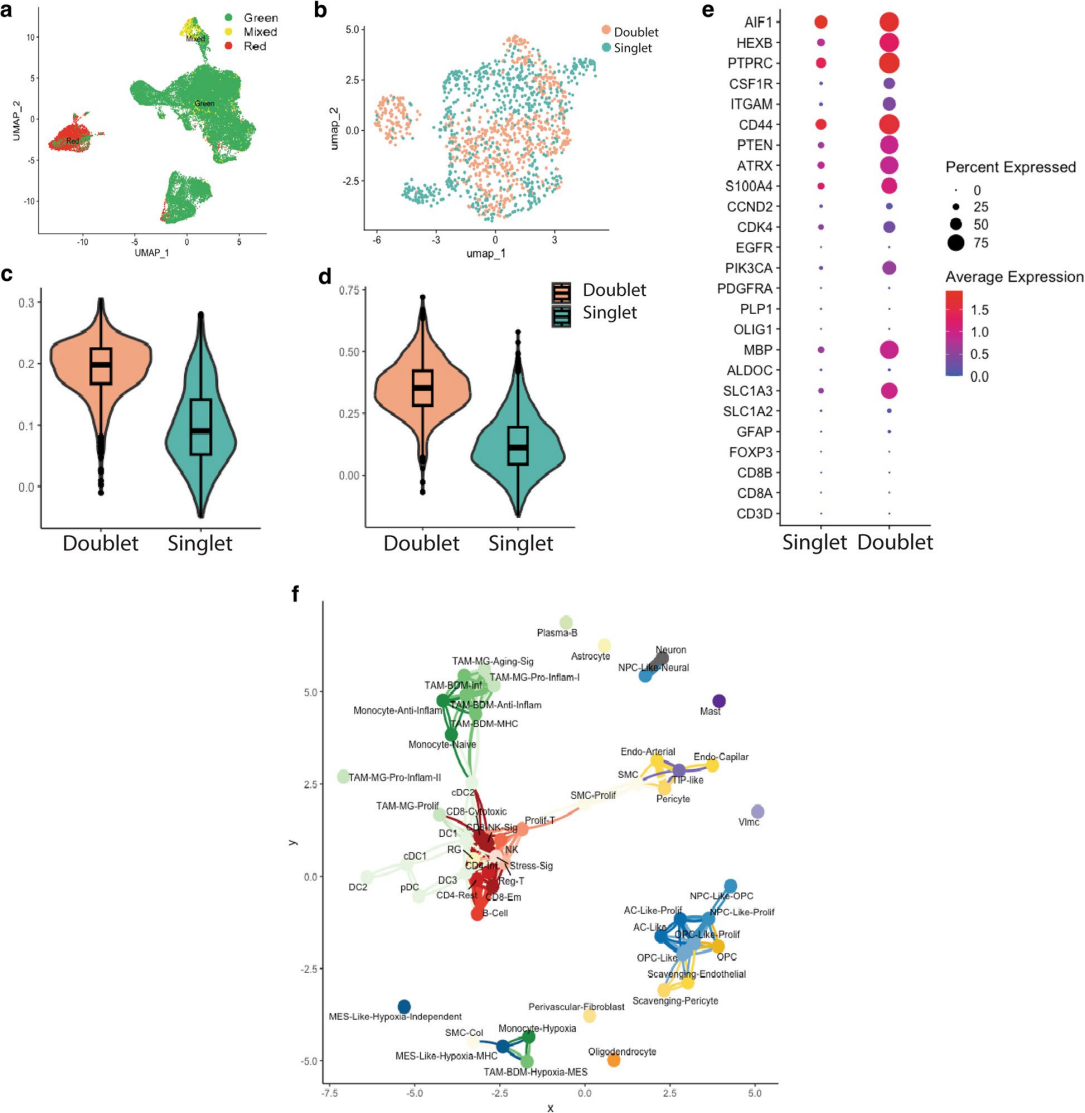
Characterization and spatial analysis of myeloid doublets. **a** UMAP representation of all cells per channel (**b**) Sub-clustering and doublet analysis of myeloid subsets showing mixed cells predicted to have a doublet profile. **c** Dotplot of average expression of key marker genes for different GB TME cell types. **d** Violin plot showing the scores of phagocytic and **e** scavenger signature scores in myeloid doublets and singlets. **f** Network graph of adjacency matrix showing inferred proximity of different cell types in spatial transcriptomics datasets, nodes represent cell types, and edges indicate significant spatial correlations between nodes. Blue colors represent different GB cell states, red colors represent immune cell types, and green colors represent TAMs and dendritic cells. Other cell types are shown in different colors

A subpopulation of cells in our dataset were both Cy5 and ZsGreen positive (Fig. 4a). scRNA-seq analysis revealed that a large fraction of these cells with double-positive signals are also transcriptionally classified as doublets during the doublet detection preprocessing step and are localized within the myeloid cells cluster (Fig. 4b). Notably, not all myeloid cells were classified as doublets. Sub-clustering analysis of the myeloid population revealed that most of the myeloid cells classified as doublets correspond to monocytes and tumor-associated macrophages (TAMs) of bone marrow origin (Additional file 1: Fig. 4a and 4b, Additional file 2: Table 6). These doublet myeloid cells show strong expression of both classical myeloid markers (*AIF1*, *HEXB*, *PTPRC*) and GB markers (*PTEN*, *ATRX*, *PIK3CA*) with no expression of classical T cell, oligodendrocyte, or astrocyte markers (Fig. 4c, Additional file 2: Table 7). The presence of myeloid cells expressing both myeloid and GB markers has been previously described [44, 45] and were shown to promote an immunosuppressive TME in GB. To further characterize the functional state of these cells, we quantified phagocytic and scavenger activity scores using established gene signatures for myeloid phagocytosis in GB [46, 47]. Myeloid doublets scored significantly higher for phagocytic and scavenger activity compared to non-doublets myeloid cells (*p* = 2.2e-16 for both comparisons) (Fig. 4d and e), indicating the activation of programs consistent with tumor-associated scavenging. While these data do not conclusively demonstrate physical engulfment of tumor cells, they support the presence of myeloid subsets exhibiting phagocytic transcriptional activity possibly engaged in clearing tumor debris. To examine whether these myeloid cells share spatial proximity with GB cells in patient samples, we leveraged our publicly available spatial transcriptomic datasets. We selected only IDHwt GBs with corresponding spatial TRC quantification [19, 48] (*n* = 5). Using minimal spanning tree proximity analysis, we found that tumor cells were in closer proximity to myeloid cells than to lymphoid or neuronal cell types (Additional file 1: Fig. 4d). A correlation analysis of cell types proximity inferred at sub-cellular level, mesenchymal-like (Mes-like) tumor cells, TAM-BDM, and monocytes associated with hypoxic regions showed strong spatial co-localization **(**Fig. 4f, Additional file 1: Fig. 4c). Our results are in line with recent studies reporting a direct interaction between myeloid cells and Mes-like GB cells [26, 49, 50]. Taken together, these results suggest that myeloid cells with doublet signature represent transcriptionally active scavenger-like TAMs, positioned in close proximity to GB cells within hypoxic niches. Although our data do not prove active engulfment, they highlight phagocytic and scavenger gene expression programs consistent with myeloid-tumor interactions in GB.

### MAF drives CAR CD8 cytotoxic T cell exhaustion

**Fig. 5:**
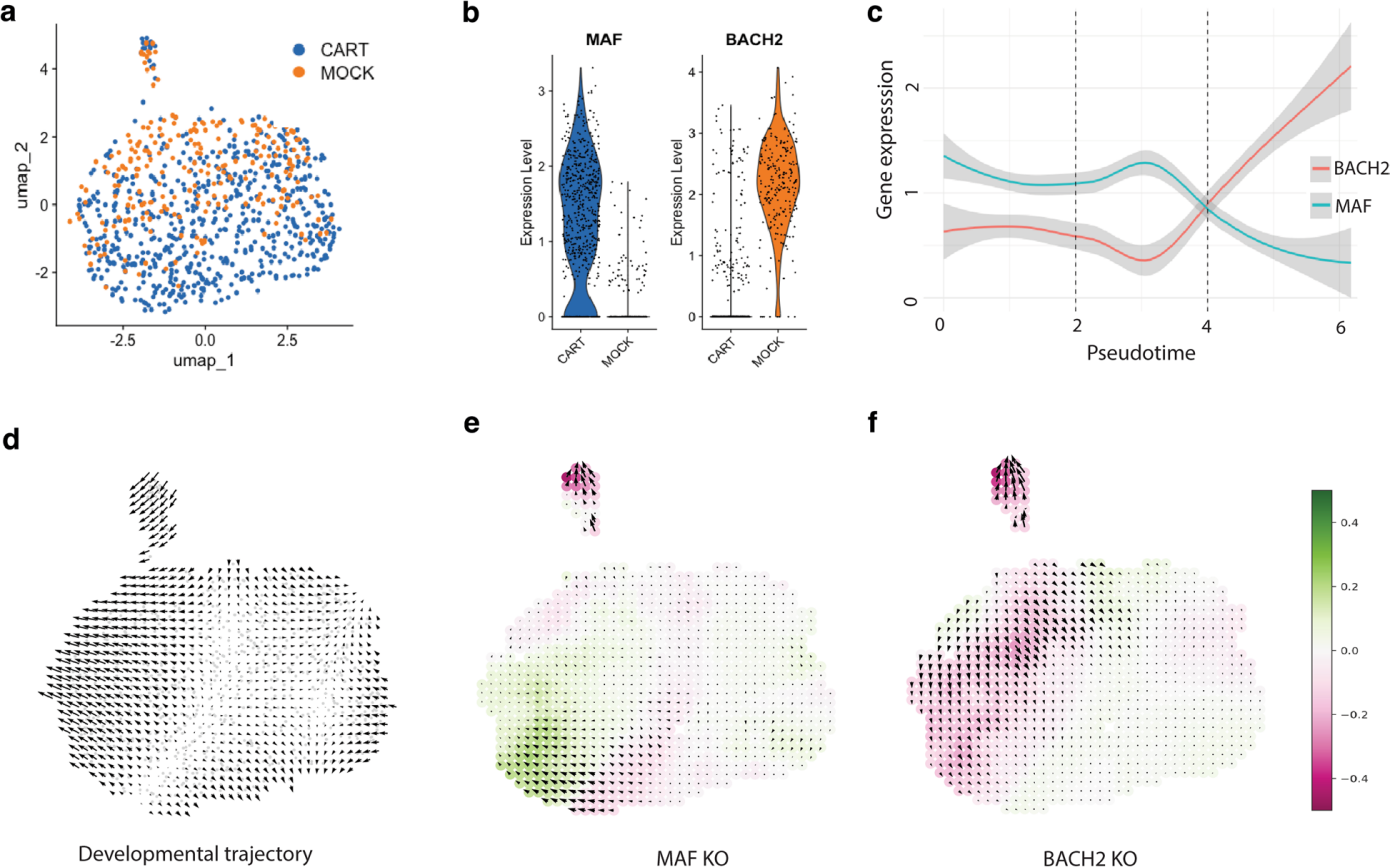
Transcriptional regulators driving CAR CD8 T cells dysregulation. **a** UMAP representation of CD8^+^ cells from CAR-T and mock groups. **b** Violin plot showing the expression levels of MAF and BACH2 genes in CD8^+^ cells.** c** Line plot showing alternate expression of BACH2 and MAF along pseudotime trajectory.** d** Normal developmental flow of CD8 T cells based on pseudotime analyses (arrows represent predicted directional shifts in cell-state probabilities derived from the GRN simulation, and the color scale indicates the magnitude of transcriptional change relative to the unperturbed state). **e** Inferred perturbation analysis showing a change of trajectory upon MAF KO (arrows indicating the direction of potential cellular state switch towards normal CD8 T cell state) and **f** and upon BACH2 KO (arrows indicating the direction of potential cellular state switch towards CD8 T cell exhaustion). The green color in **e** and **f** shows similarity between the developmental trajectory and perturbation vectors while pink color shows dissimilarity between the vectors. The deeper the color the stronger the similarity or dissimilarity

To understand the key regulators of CAR-T cell dysfunction, we extracted only CD8 T cells from our scRNA-seq dataset representing CD8 T cells in CAR-T and Mock groups. We reconstructed the gene regulatory networks (GRN) of these CD8 subsets using CellOracle [51] and identified the most highly active transcription factors (TFs) associated with CAR-T and Mock groups (Fig. 5a). TFs with the highest centrality scores were inferred to control cellular functions such as cytotoxicity and exhaustion, whereas a high betweenness centrality score identified essential TFs essential for the flow of transcriptional information within the GRN. Among the top 30 TFs with the highest degree centrality score, *BACH2* and *MAF* have the highest betweenness centrality in CD8 CAR-T cells when compared to CD8 Mock T cells (Additional file 1: Fig. 5a), indicating that these TFs are central to the maintenance and stabilization of CD8 T cell functional states. Since *BACH2* and *MAF* are inferred to control the CD8 functional state, we examined their expression in CD8 T cell subsets of our scRNA-seq data. We found the expression of *BACH2* and *MAF* to be consistent with their inferred activity (Fig. 5b). *BACH2* is highly expressed in CD8 Mock T cells while *MAF* is expressed mainly by the CD8 CAR-T cells, indicating an alternate expression pattern between the different groups. Pseudotime analysis of the expression of *MAF* and *BACH2* indicated an alternate expression of these genes (Fig. 5c, Additional file 1: Fig. 5b). While *MAF* is upregulated in CD8 T cells, *BACH2* is downregulated and vice versa (Additional file 1: Fig. 5c). This reflects distinct regulatory influences rather than strict developmental transitions. We asked if *MAF* and *BACH2* expression dynamics are present in T cells within the patient GB samples and other CAR-T cells. Interestingly, we found that while *MAF* was found to be upregulated in exhausted CD8 T cells in GB patient samples [52] (Additional file 1: Fig. 5d) and CAR constructs with an exhaustion phenotype in mouse models [53] (Additional file 1: Fig. 5e), *BACH2* is elevated in naïve CD8 T cells in patient samples and in normal CD8 T cells used as control while comparing several CAR-T constructs in mouse GB models [53, 54]. To further understand the effect of these TFs in driving and maintaining CD8 T cell exhaustion, we performed in silico perturbation of *MAF* and *BACH2* using our scRNA-seq dataset and the GRNs constructed using CellOracle. We inferred the normal developmental trajectory by pseudotime analysis. This inference analysis indicates potentially two different developmental pathways or cell states within our mixed CD8 populations (Fig. 5d). Each of the developmental axes correlates with either CAR-T or Mock groups as shown in (Fig. 5a). Upon the simulation of *MAF* knock-out (KO), the model predicts a shift toward a less exhaustion-like state (from CAR-T to the Mock group) (Fig. 5e), while upon simulation of *BACH2* KO, the model shows a shift towards a regulatory state associated with CAR CD8 cell exhaustion (from Mock to CAR-T group) (Fig. 5f). These results indicate that the activity of *MAF* promotes a shift towards exhaustion-like transcriptional programs, whereas the activity of *BACH2* supports the maintenance of non-exhausted CD8 T cell state. Importantly, these conclusions only reflect predicted regulatory effects from in silico perturbation, rather than direct state transitions.

## Discussion

Resistance to CAR-T cell therapy in GB arises from tumor-intrinsic or extrinsic factors such as an immunosuppressive microenvironment. Using a systems biology approach, we integrated PIC-seq and spatial transcriptomics to investigate CAR-T cell dysfunction. Comparative analysis of CAR-T and Mock T cells revealed distinct gene regulatory programs in the GB TME. Neocortical brain slices recapitulated the cellular architecture of patient tumors and supported tumor progression. Both Mock and CAR-T cells interacted with tumor and stromal components in the neuronal microenvironment. Interactions between CAR-T cells and myeloid cells provided an immunosuppressive niche that impaired CD8 CAR-T cell function. A spatially localized myeloid subpopulation with the expression of phagocytic genes was enriched near tumor cells. We identified *MAF* and *BACH2* as key transcriptional regulators, with *MAF* driving CAR-T cells to a dysfunctional state and *BACH2* maintaining normal T cell developmental programs.

CAR-T cell therapy has been successful in other malignancies but has so far failed to cure GB patients. The reasons for the low efficacy in GB and other solid tumors have not been fully understood [1]. Recent results from early clinical trials reported a rapid but shortened antitumor effect in recurrent GB [3, 4]. Unlike the results from human clinical trials, CAR-T cell therapy in murine models shows a remarkably positive and sustained response across different treatment strategies [14, 20, 21, 55]. Similar to human clinical trials results and unlike in mouse models, we show that CAR-T cells demonstrated a strong initial cytotoxic effect but were unable to maintain sustained cytotoxic activity in our model. Further analysis revealed the activation of CAR CD8 exhaustion-related signaling pathways and myeloid cell mobilization in the hypoxic tumor niche, leading to a suppressive microenvironment and activation of CD8 T cell exhaustion-like programs.

Exhausted cytotoxic T cells represent a distinct phenotypic state of T cells following intense activation in the context of chronic infections or cancer [56]. While initially responding to antigens, these T cells experience a progressive loss of function, marked by reduced cytotoxicity and diminished cytokine production [57]. This dysfunctional state is a consequence of multiple factors, including impaired regulation, an immunosuppressive TME, and tumor-specific adaptations. These circumstances collectively drive T cells into a state of exhaustion and dysfunction, hindering their ability to effectively eliminate the tumor cells [57, 58]. Crosstalk between myeloid cells and T cells in GB is known to promote immunosuppression and T cell dysfunction [26, 59, 60]. The dysfunctionality of CD8 T cells which seems to result from interaction with myeloid cells of the TME highlights the important role of the TME in driving and maintaining T cell exhaustion. In line with our observations, CD8 T cells have been reported to physically interact with TAMs forming a long-lasting synapse that initiates and maintains CD8 exhaustion [59, 61]. Interestingly, the initiation of CD8 T cell exhaustion by TAMs cells that is accelerated in the hypoxic tumor niche, as reported by Kersten et al., aligns with our observation of spatial proximity of monocytes, mes-like GB cells, and TAMs in a hypoxic environment. We have previously shown that myeloid cells are spatially located in tumor-T cell neighborhoods [26]. In addition, our study further highlights the specific ligands-receptors involved in this interaction. Although further research is needed to fully describe and validate the signaling between TAMs and CD8 T cells, we identified the CLEC pathway as a potential target in interfering myeloid-GB interaction driving CD8 T cell dysfunction and subsequent hyporesponsiveness.

In recent studies, myeloid-tumor doublets have also been identified and linked to immunosuppressive function in GB, however their spatial localization remains unclear [26, 44, 49]. Our results indicating the spatial proximity of myeloid cells with Mes-like GB cells within the hypoxic tumor niche and having enhanced phagocytic and scavenger signatures may indicate the clearance of apoptotic GB cells to avoid the engulfment and processing of the GB cells by antigen-presenting cells (APCs). This prevents the APCs from presenting tumor antigens to CAR-T cells, hence contributing to the rapid exhaustion of the CAR-T cells and sustaining an immunosuppressed TME. Both our data and previous studies [19, 44, 49] indicate that the engulfment of GB cells by myeloid cells plays an important role in shaping the GB microenvironment by driving immunosuppression. More experimental studies are needed to fully understand the nature and activity of these myeloid-tumor doublets.

We identified *MAF* and *BACH2* as key transcriptional regulators linked to dysfunctional CD8 T-cell states. While *MAF* is upregulated in the CAR CD8 T cells, *BACH2* is upregulated in Mock CD8 T cells. *MAF*, a member of the AP-1 (Activator Protein-1) family, is induced in tumor-associated CD8 T cells downstream of TGF-β and IL-6 and promotes programs associated with exhaustion [62–64]. By contrast, *BACH2* functions primarily as a transcriptional repressor that restrains terminal effector differentiation and favors stem-like and memory T-cell programs [65–67]. Through the inhibition of IRF4- and AP-1–driven gene expression, *BACH2* helps maintain a progenitor-like pool with long-term persistence rather than sustaining immediate effector activity [65, 68]. Consistent with this, since *BACH2* has been implicated in memory formation, its downregulation is expected to reduce memory potential and permit progression toward terminal effector and dysfunctional states as recently reported [54, 67, 69–71]. *BACH2* can bind the *MAF* locus and, together with *MAF*, form complexes that dampen AP-1–dependent transcription, thereby buffering excessive T cell activation [65, 72, 73]. Thus, loss or low activity of *BACH2* is consistent with stronger CAR-driven activation and differentiation pushing the cells towards precursor exhaustion-like state.

## Limitations of the study

The CAR construct used in this study was delivered via mRNA electroporation, which offers high transfection efficiency but results in transient protein expression. Alternative delivery methods that support prolonged CAR expression may yield different outcomes. Additionally, there was an imbalance in T cell subpopulations between CAR-T and Mock-treated slices, with CD4 and regulatory T cells absent in the Mock group. Given the known importance of CD4 T cells in sustaining antitumor responses and the differential susceptibility of CD8 T cells to exhaustion, this discrepancy may influence comparative interpretations. Prior studies have shown that CD4 CAR-T cells can mediate more durable responses, whereas CD8 T cells may dampen CD4-driven efficacy in GB. Therefore, future designs of CAR-T studies and therapies should consider the CD4:CD8 ratio as a critical variable in optimizing therapeutic efficacy. Finally, some of our conclusions are derived from computational inference analyses. While these observations are strongly indicative, definitive confirmations will require functional validations in future studies.

## Conclusions

Our study integrates ex vivo brain-slice modeling with single-cell and spatial transcriptomics to characterize microenvironmental and cell-intrinsic features associated with the rapid attenuation of CAR-T activity in GB. We highlight how myeloid-T cell and myeloid-tumor interactions within hypoxic, mesenchymal-like niches, together with coordinated CD8 T cell exhaustion-like transcriptional programs, contribute to an immunosuppressive microenvironment. The signaling pathways and transcriptional regulators identified are targetable candidates for improving CAR-T cells engineering strategies for enhanced therapeutic durability.

## Supplementary Information

Additional file 1.

Additional file 2.

Additional file 3.

## Data Availability

All data are included in the manuscript and the supplementary files. Data used in the analyses in this paper is available from the corresponding authors upon reasonable request.

## Abbreviations

AP-1: Activator protein-1
APCs: Antigen-presenting cells
BACH2: BTB Domain and CNC Homolog 2
BP: Biological process
CAR: Chimeric antigen receptor
CLEC: C-type lectin-like receptor
CNV: Copy number variation
CTFR: Cell trace far red
DEGs: Differentially expressed genes
DFIB: DAPI-containing FACS incubation buffer
DPI: Day post-tumor injection
EGFRvIII: Epidermal growth factor receptor deletion mutant variant III
FACS: Fluorescence-activated cell sorting
GB: Glioblastoma
GBmap: Glioblastoma map
GFP: Green fluorescent protein
GO: Gene Ontology
GRN: Gene regulatory network
HER2: Human epidermal growth factor receptor 2
HMM: Hidden Markov model
HMOX1: Heme Oxygenase-1
IL: Interleukin
IL-13Rα2: Interleukin-13 receptor alpha 2
KO: Knock out
LAG-3: Lymphocyte activation gene-3
MAF: Musculoaponeurotic fibrosarcoma
MNN: Mutual nearest neighbors
mRNA: Messenger ribonucleic acid
MsigDB: Molecular Signatures Database
NKG2D: Natural killer group 2D
PBMC: Peripheral Blood Mononuclear Cell
PCA: Principal component analysis
PD-1: Programmed Cell Death-1
PDX: Patient-derived xenograft
PHATE: Potential of Heat-diffusion for Affinity-based Transition Embedding
PIC-seq: Physically interacting cells sequencing
scRNA-seq: Single-cell RNA sequencing
SNN: Shared nearest neighbor
SNT: Simple neurite tracer
TAMs: Tumor-associated macrophages
TAM-BDM: Tumor-associated macrophages–bone marrow derived
TRC: T cell receptors
TFs: Transcription factors
TGF-β: Transforming growth factor-beta
TIM-3: T cell Immunoglobulin and Mucin domain-containing protein-3
TME: Tumor microenvironment
UMAP: Uniform manifold approximation and projection

## References

[1] Majzner RG, Mackall CL. Clinical lessons learned from the first leg of the CAR T cell journey. Nat Med. 2019;25(9):1341–55. 31501612 10.1038/s41591-019-0564-6

[2] Guzman G, Pellot K, Reed MR, Rodriguez A. CAR T-cells to treat brain tumors. Brain Res Bull. 2023;196:76–98. 36841424 10.1016/j.brainresbull.2023.02.014

[3] Bagley SJ, Logun M, Fraietta JA, Wang X, Desai AS, Bagley LJ, et al. Intrathecal bivalent CAR T cells targeting EGFR and IL13Rα2 in recurrent glioblastoma: phase 1 trial interim results. Nat Med. 2024;30(5):1320–9. 38480922 10.1038/s41591-024-02893-z PMC13123313

[4] Choi BD, Gerstner ER, Frigault MJ, Leick MB, Mount CW, Balaj L, et al. Intraventricular CARv3-TEAM-E T cells in recurrent glioblastoma. N Engl J Med. 2024;390(14):1290–8. 38477966 10.1056/NEJMoa2314390 PMC11162836

[5] O’Rourke DM, Nasrallah MP, Desai A, Melenhorst JJ, Mansfield K, Morrissette JJD, et al. A single dose of peripherally infused EGFRvIII-directed CAR T cells mediates antigen loss and induces adaptive resistance in patients with recurrent glioblastoma. Sci Transl Med. 2017. 10.1126/scitranslmed.aaa0984. 28724573 10.1126/scitranslmed.aaa0984 PMC5762203

[6] Bagley SJ, Binder ZA, Lamrani L, Marinari E, Desai AS, Nasrallah MP, et al. Repeated peripheral infusions of anti-EGFRvIII CAR T cells in combination with pembrolizumab show no efficacy in glioblastoma: a phase 1 trial. Nat Cancer. 2024;5(3):517–31. 38216766 10.1038/s43018-023-00709-6

[7] Yabo YA, Niclou SP, Golebiewska A. Cancer cell heterogeneity and plasticity: a paradigm shift in glioblastoma. Neuro Oncol. 2022;24(5):669–82. 34932099 10.1093/neuonc/noab269 PMC9071273

[8] Park DH, Liaw K, Bhojnagarwala P, Zhu X, Choi J, Ali AR, et al. Multivalent in vivo delivery of DNA-encoded bispecific T cell engagers effectively controls heterogeneous GBM tumors and mitigates immune escape. Mol Ther Oncolytics. 2023;28:249–63. 36915911 10.1016/j.omto.2023.02.004 PMC10006507

[9] Bagley SJ, Desai AS, Fraietta JA, Silverbush D, Chafamo D, Freeburg NF, et al. Intracerebroventricular bivalent CAR T cells targeting EGFR and IL-13Rα2 in recurrent glioblastoma: a phase 1 trial. Nat Med. 2025;31(8):2778–87. 40451950 10.1038/s41591-025-03745-0 PMC12715680

[10] Prajapati K, Perez C, Rojas LBP, Burke B, Guevara-Patino JA. Functions of NKG2D in CD8+ T cells: an opportunity for immunotherapy. Cell Mol Immunol. 2018;15(5):470–9. 29400704 10.1038/cmi.2017.161 PMC6068164

[11] Weiss T, Weller M, Guckenberger M, Sentman CL, Roth P. NKG2D-based CAR T cells and radiotherapy exert synergistic efficacy in glioblastoma. Cancer Res. 2018;78(4):1031–43. 29222400 10.1158/0008-5472.CAN-17-1788

[12] Yang D, Sun B, Dai H, Li W, Shi L, Zhang P, et al. T cells expressing NKG2D chimeric antigen receptors efficiently eliminate glioblastoma and cancer stem cells. J Immunother Cancer. 2019;7(1):171. 31288857 10.1186/s40425-019-0642-9 PMC6617951

[13] Weiss T, Schneider H, Silginer M, Steinle A, Pruschy M, Polić B, et al. NKG2D-dependent antitumor effects of chemotherapy and radiotherapy against glioblastoma. Clin Cancer Res. 2018;24(4):882–95. 29162646 10.1158/1078-0432.CCR-17-1766

[14] Meister H, Look T, Roth P, Pascolo S, Sahin U, Lee S, et al. Multifunctional mRNA-based CAR T cells display promising antitumor activity against glioblastoma. Clin Cancer Res. 2022;28(21):4747–56. 36037304 10.1158/1078-0432.CCR-21-4384

[15] Look T, Sankowski R, Bouzereau M, Fazio S, Sun M, Buck A, et al. CAR T cells, CAR NK cells, and CAR macrophages exhibit distinct traits in glioma models but are similarly enhanced when combined with cytokines. Cell Rep Med. 2025;6(2):101931. 39889712 10.1016/j.xcrm.2025.101931 PMC11866521

[16] Bagley SJ, Desai AS, Linette GP, June CH, O’Rourke DM. CAR T-cell therapy for glioblastoma: recent clinical advances and future challenges. Neuro Oncol. 2018;20(11):1429–38. 29509936 10.1093/neuonc/noy032 PMC6176794

[17] Blank CU, Haining WN, Held W, Hogan PG, Kallies A, Lugli E, et al. Defining “T cell exhaustion.” Nat Rev Immunol. 2019;19(11):665–74. 31570879 10.1038/s41577-019-0221-9 PMC7286441

[18] Cai L, Li Y, Tan J, Xu L, Li Y. Targeting LAG-3, TIM-3, and TIGIT for cancer immunotherapy. J Hematol Oncol. 2023;16(1):101. 37670328 10.1186/s13045-023-01499-1 PMC10478462

[19] Ravi VM, Will P, Kueckelhaus J, Sun N, Joseph K, Salié H, et al. Spatially resolved multi-omics deciphers bidirectional tumor-host interdependence in glioblastoma. Cancer Cell. 2022;40(6):639-655.e13. 35700707 10.1016/j.ccell.2022.05.009

[20] Agliardi G, Liuzzi AR, Hotblack A, De Feo D, Núñez N, Stowe CL, et al. Intratumoral IL-12 delivery empowers CAR-T cell immunotherapy in a pre-clinical model of glioblastoma. Nat Commun. 2021;12(1):444. 33469002 10.1038/s41467-020-20599-x PMC7815781

[21] Schmidts A, Srivastava AA, Ramapriyan R, Bailey SR, Bouffard AA, Cahill DP, et al. Tandem chimeric antigen receptor (CAR) T cells targeting EGFRvIII and IL-13Rα2 are effective against heterogeneous glioblastoma. Neuro-Oncol Adv. 2023;5(1):vdac185. 10.1093/noajnl/vdac185 PMC9896600 36751672

[22] Choe JH, Watchmaker PB, Simic MS, Gilbert RD, Li AW, Krasnow NA, et al. SynNotch-CAR T cells overcome challenges of specificity, heterogeneity, and persistence in treating glioblastoma. Sci Transl Med. 2021. 10.1126/scitranslmed.abe7378. 33910979 10.1126/scitranslmed.abe7378 PMC8362330

[23] Hyrenius-Wittsten A, Su Y, Park M, Garcia JM, Alavi J, Perry N, et al. SynNotch CAR circuits enhance solid tumor recognition and promote persistent antitumor activity in mouse models. Sci Transl Med. 2021;13(591):eabd8836. 10.1126/scitranslmed.abd8836 PMC8594452 33910981

[24] Zhang J, Straehle J, Joseph K, Neidert N, Behringer S, Göldner J, et al. Isolation and profiling of viable tumor cells from human ex vivo glioblastoma cultures through single-cell transcriptomics. STAR Protocols. 2023;4(3):102383. 37393609 10.1016/j.xpro.2023.102383 PMC10328984

[25] Giladi A, Cohen M, Medaglia C, Baran Y, Li B, Zada M, et al. Dissecting cellular crosstalk by sequencing physically interacting cells. Nat Biotechnol. 2020;38(5):629–37. 32152598 10.1038/s41587-020-0442-2

[26] Ravi VM, Neidert N, Will P, Joseph K, Maier JP, Kückelhaus J, et al. T-cell dysfunction in the glioblastoma microenvironment is mediated by myeloid cells releasing interleukin-10. Nat Commun. 2022;13(1):925. 35177622 10.1038/s41467-022-28523-1 PMC8854421

[27] Moon KR, van Dijk D, Wang Z, Gigante S, Burkhardt DB, Chen WS, et al. Visualizing structure and transitions in high-dimensional biological data. Nat Biotechnol. 2019;37(12):1482–92. 31796933 10.1038/s41587-019-0336-3 PMC7073148

[28] Street K, Risso D, Fletcher RB, Das D, Ngai J, Yosef N, et al. Slingshot: cell lineage and pseudotime inference for single-cell transcriptomics. BMC Genomics. 2018;19(1):477. 29914354 10.1186/s12864-018-4772-0 PMC6007078

[29] McGinnis CS, Murrow LM, Gartner ZJ. Doubletfinder: doublet detection in single-cell RNA sequencing data using artificial nearest neighbors. Cell Syst. 2019;8(4):329-337.e4. 30954475 10.1016/j.cels.2019.03.003 PMC6853612

[30] Wherry EJ, Ha S-J, Kaech SM, Haining WN, Sarkar S, Kalia V, et al. Molecular signature of CD8+ T cell exhaustion during chronic viral infection. Immunity. 2007;27(4):670–84. 17950003 10.1016/j.immuni.2007.09.006

[31] Kim T-S, Shin E-C. The activation of bystander CD8+ T cells and their roles in viral infection. Exp Mol Med. 2019;51(12):1–9. 31827070 10.1038/s12276-019-0316-1 PMC6906361

[32] Hao Y, Hao S, Andersen-Nissen E, Mauck WM, Zheng S, Butler A, et al. Integrated analysis of multimodal single-cell data. Cell. 2021;184(13):3573–87. 34062119 10.1016/j.cell.2021.04.048 PMC8238499

[33] Ruiz-Moreno C, Marco Salas S, Samuelsson E, Minaeva M, Ibarra I, Grillo M, et al. Charting the single-cell and spatial landscape of IDH-wild-type glioblastoma with GBmap. Neuro Oncol. 2025;27(9):2281–95. 10.1093/neuonc/noaf113 PMC12526130 40312969

[34] Wang D, Aguilar B, Starr R, Alizadeh D, Brito A, Sarkissian A, et al. Glioblastoma-targeted CD4+ CAR T cells mediate superior antitumor activity. JCI Insight. 2018. 10.1172/jci.insight.99048. 29769444 10.1172/jci.insight.99048 PMC6012522

[35] Vardhana SA, Hwee MA, Berisa M, Wells DK, Yost KE, King B, et al. Impaired mitochondrial oxidative phosphorylation limits the self-renewal of T cells exposed to persistent antigen. Nat Immunol. 2020;21(9):1022–33. 32661364 10.1038/s41590-020-0725-2 PMC7442749

[36] Bengsch B, Johnson AL, Kurachi M, Odorizzi PM, Pauken KE, Attanasio J, et al. Bioenergetic insufficiencies due to metabolic alterations regulated by the inhibitory receptor PD-1 are an early driver of CD8(+) T cell exhaustion. Immunity. 2016;45(2):358–73. 27496729 10.1016/j.immuni.2016.07.008 PMC4988919

[37] Scharping NE, Menk AV, Moreci RS, Whetstone RD, Dadey RE, Watkins SC, et al. The tumor microenvironment represses T cell mitochondrial biogenesis to drive intratumoral T cell metabolic insufficiency and dysfunction. Immunity. 2016;45(2):374–88. 27496732 10.1016/j.immuni.2016.07.009 PMC5207350

[38] Buck MD, O’Sullivan D, Pearce EL. T cell metabolism drives immunity. J Exp Med. 2015;212(9):1345–60. 26261266 10.1084/jem.20151159 PMC4548052

[39] Omilusik KD, Best JA, Yu B, Goossens S, Weidemann A, Nguyen JV, et al. Transcriptional repressor ZEB2 promotes terminal differentiation of CD8+ effector and memory T cell populations during infection. J Exp Med. 2015;212(12):2027–39. 26503445 10.1084/jem.20150194 PMC4647262

[40] Rutishauser RL, Martins GA, Kalachikov S, Chandele A, Parish IA, Meffre E, et al. Transcriptional repressor Blimp-1 promotes CD8(+) T cell terminal differentiation and represses the acquisition of central memory T cell properties. Immunity. 2009;31(2):296–308. 19664941 10.1016/j.immuni.2009.05.014 PMC2783637

[41] Jung I-Y, Narayan V, McDonald S, Rech AJ, Bartoszek R, Hong G, et al. BLIMP1 and NR4A3 transcription factors reciprocally regulate antitumor CAR T cell stemness and exhaustion. Sci Transl Med. 2022;14(670):eabn7336. 36350986 10.1126/scitranslmed.abn7336 PMC10257143

[42] Liberzon A, Birger C, Thorvaldsdóttir H, Ghandi M, Mesirov JP, Tamayo P. The molecular signatures database (MSigDB) hallmark gene set collection. Cell Syst. 2015;1(6):417–25. 26771021 10.1016/j.cels.2015.12.004 PMC4707969

[43] Jin S, Guerrero-Juarez CF, Zhang L, Chang I, Ramos R, Kuan C-H, et al. Inference and analysis of cell-cell communication using Cell Chat. Nat Commun. 2021;12(1):1088. 33597522 10.1038/s41467-021-21246-9 PMC7889871

[44] Wu M, Wu L, Wu W, Zhu M, Li J, Wang Z, et al. Phagocytosis of glioma cells enhances the immunosuppressive phenotype of bone marrow-derived macrophages. Cancer Res. 2023;83(5):771–85. 36622331 10.1158/0008-5472.CAN-22-1570 PMC9978884

[45] Cao M-F, Chen L, Dang W-Q, Zhang X-C, Zhang X, Shi Y, et al. Hybrids by tumor-associated macrophages × glioblastoma cells entail nuclear reprogramming and glioblastoma invasion. Cancer Lett. 2019;442:445–52. 30472185 10.1016/j.canlet.2018.11.016

[46] Yabo YA, Moreno-Sanchez PM, Pires-Afonso Y, Kaoma T, Nosirov B, Scafidi A, et al. Glioblastoma-instructed microglia transition to heterogeneous phenotypic states with phagocytic and dendritic cell-like features in patient tumors and patient-derived orthotopic xenografts. Genome Med. 2024;16(1):51. 38566128 10.1186/s13073-024-01321-8 PMC10988817

[47] Miller TE, El Farran CA, Couturier CP, Chen Z, D’Antonio JP, Verga J, et al. Programs, origins and immunomodulatory functions of myeloid cells in glioma. Nature. 2025;640(8060):1072–82. 40011771 10.1038/s41586-025-08633-8 PMC12018266

[48] Benotmane JK, Kueckelhaus J, Will P, Zhang J, Ravi VM, Joseph K, et al. High-sensitive spatially resolved T cell receptor sequencing with SPTCR-seq. Nat Commun. 2023;14(1):7432. 37973846 10.1038/s41467-023-43201-6 PMC10654577

[49] Gangoso E, Southgate B, Bradley L, Rus S, Galvez-Cancino F, McGivern N, et al. Glioblastomas acquire myeloid-affiliated transcriptional programs via epigenetic immunoediting to elicit immune evasion. Cell. 2021;184(9):2454-2470.e26. 33857425 10.1016/j.cell.2021.03.023 PMC8099351

[50] Hara T, Chanoch-Myers R, Mathewson ND, Myskiw C, Atta L, Bussema L, et al. Interactions between cancer cells and immune cells drive transitions to mesenchymal-like states in glioblastoma. Cancer Cell. 2021;39(6):779-792.e11. 34087162 10.1016/j.ccell.2021.05.002 PMC8366750

[51] Kamimoto K, Stringa B, Hoffmann CM, Jindal K, Solnica-Krezel L, Morris SA. Dissecting cell identity via network inference and in silico gene perturbation. Nature. 2023;614(7949):742–51. 36755098 10.1038/s41586-022-05688-9 PMC9946838

[52] Dobersalske C, Rauschenbach L, Hua Y, Berliner C, Steinbach A, Grüneboom A, et al. Cranioencephalic functional lymphoid units in glioblastoma. Nat Med. 2024;30(10):2947–56. 39085419 10.1038/s41591-024-03152-x PMC11485206

[53] Haydar D, Ibañez-Vega J, Crawford JC, Chou C-H, Guy CS, Meehl M, et al. CAR T-cell design-dependent remodeling of the brain tumor immune microenvironment modulates tumor-associated macrophages and anti-glioma activity. Cancer Res Commun. 2023;3(12):2430–46. 37971169 10.1158/2767-9764.CRC-23-0424 PMC10689147

[54] Hu T, Zhu Z, Luo Y, Wizzard S, Hoar J, Shinde SS, et al. BACH2 dosage establishes the hierarchy of stemness and fine-tunes antitumor immunity in CAR T cells. Nat Immunol. 2026;27(3):425–35. 10.1038/s41590-025-02388-0 PMC12956562 41545542

[55] Zhou S, Huang Y, Chen Y, Liu Y, Xie L, You Y, et al. Reprogramming systemic and local immune function to empower immunotherapy against glioblastoma. Nat Commun. 2023;14(1):435. 36702831 10.1038/s41467-023-35957-8 PMC9880004

[56] McLane LM, Abdel-Hakeem MS, Wherry EJ. CD8 T cell exhaustion during chronic viral infection and cancer. Annu Rev Immunol. 2019;37:457–95. 30676822 10.1146/annurev-immunol-041015-055318

[57] Gumber D, Wang LD. Improving CAR-T immunotherapy: overcoming the challenges of T cell exhaustion. EBioMedicine. 2022;77:103941. 35301179 10.1016/j.ebiom.2022.103941 PMC8927848

[58] Gebhardt T, Park SL, Parish IA. Stem-like exhausted and memory CD8+ T cells in cancer. Nat Rev Cancer. 2023;23(11):780–98. 37821656 10.1038/s41568-023-00615-0

[59] Kersten K, Hu KH, Combes AJ, Samad B, Harwin T, Ray A, et al. Spatiotemporal co-dependency between macrophages and exhausted CD8+ T cells in cancer. Cancer Cell. 2022;40(6):624-638.e9. 35623342 10.1016/j.ccell.2022.05.004 PMC9197962

[60] Watowich MB, Gilbert MR, Larion M. T cell exhaustion in malignant gliomas. Trends Cancer. 2023;9(4):270–92. 36681605 10.1016/j.trecan.2022.12.008 PMC10038906

[61] Peranzoni E, Lemoine J, Vimeux L, Feuillet V, Barrin S, Kantari-Mimoun C, et al. Macrophages impede CD8 T cells from reaching tumor cells and limit the efficacy of anti-PD-1 treatment. Proc Natl Acad Sci USA. 2018;115(17):E4041–50. 29632196 10.1073/pnas.1720948115 PMC5924916

[62] Imbratta C, Hussein H, Andris F, Verdeil G. c-MAF, a Swiss army knife for tolerance in lymphocytes. Front Immunol. 2020;11:206. 32117317 10.3389/fimmu.2020.00206 PMC7033575

[63] Xu J, Yang Y, Qiu G, Lal G, Wu Z, Levy DE, et al. c-Maf regulates IL-10 expression during Th17 polarization. J Immunol. 2009;182(10):6226–36. 19414776 10.4049/jimmunol.0900123 PMC2834209

[64] Rutz S, Noubade R, Eidenschenk C, Ota N, Zeng W, Zheng Y, et al. Transcription factor c-Maf mediates the TGF-β-dependent suppression of IL-22 production in T(H)17 cells. Nat Immunol. 2011;12(12):1238–45. 22001828 10.1038/ni.2134

[65] Roychoudhuri R, Clever D, Li P, Wakabayashi Y, Quinn KM, Klebanoff CA, et al. BACH2 regulates CD8(+) T cell differentiation by controlling access of AP-1 factors to enhancers. Nat Immunol. 2016;17(7):851–60. 27158840 10.1038/ni.3441 PMC4918801

[66] Roychoudhuri R, Hirahara K, Mousavi K, Clever D, Klebanoff CA, Bonelli M, et al. BACH2 represses effector programs to stabilize T(reg)-mediated immune homeostasis. Nature. 2013;498(7455):506–10. 23728300 10.1038/nature12199 PMC3710737

[67] Hu G, Chen J. A genome-wide regulatory network identifies key transcription factors for memory CD8^+^ T-cell development. Nat Commun. 2013;4:2830. 24335726 10.1038/ncomms3830 PMC3999894

[68] Weng X, Zheng M, Liu Y, Lou G. The role of Bach2 in regulating CD8 + T cell development and function. Cell Commun Signal. 2024;22(1):169. 38459508 10.1186/s12964-024-01551-8 PMC10921639

[69] Yao C, Lou G, Sun H-W, Zhu Z, Sun Y, Chen Z, et al. BACH2 enforces the transcriptional and epigenetic programs of stem-like CD8+ T cells. Nat Immunol. 2021;22(3):370–80. 33574619 10.1038/s41590-021-00868-7 PMC7906956

[70] Chang T-C, Heard A, Lattin J, Warrington JM, Barrett A, Landmann JH, et al. BACH2 regulates T cell lineage state to enhance CAR T cell function. Nat Immunol. 2026;27(3):413–24. 10.1038/s41590-025-02391-5 PMC12979190 41545540

[71] Conti AG, Evans AC, von Linde T, Deguit CDT, Whiteside SK, Wesolowski AJ, et al. Fine-tuning BACH2 dosage balances stemness and effector function to enhance antitumor T cell therapy. Nat Immunol. 2026;27(3):436–51. 10.1038/s41590-025-02389-z PMC12956556 41545541

[72] Liu G, Liu F. Bach2: a key regulator in Th2-related immune cells and Th2 immune response. J Immunol Res. 2022;2022:2814510. 35313725 10.1155/2022/2814510 PMC8934237

[73] Kamio T, Toki T, Kanezaki R, Sasaki S, Tandai S, Terui K, et al. B-cell-specific transcription factor BACH2 modifies the cytotoxic effects of anticancer drugs. Blood. 2003;102(9):3317–22. 12829606 10.1182/blood-2002-12-3656

