# Supplementary material for: Myeloid-derived immunosuppression of chimeric antigen receptor T cells in the neuronal microenvironment of glioblastoma
Source: BMC Med. 2026 Mar 13;24:212. doi: 10.1186/s12916-026-04783-2 (PMC13063468; doi:10.1186/s12916-026-04783-2)
Supplement: Supplementary file 1 — Additional file 1. [file 12916_2026_4783_MOESM1_ESM.docx]

**Additional file 1**

**Myeloid-derived immunosuppression of Chimeric Antigen Receptor T cells in the neuronal microenvironment of Glioblastoma**

*Junyi Zhang*^1,2,3#^*, Jasmin von Ehr*^1,2,3#^*, Thomas Look*^4^*, Jasim Kada Benotmane*^1,2,3,5^*, Nicolas Neidert*^1,2,3^*, Jan Kueckelhaus*^1,2,3,5^*, Tobias Weiss*^4^*, Dieter Henrik Heiland*^1,2,3,5,6,7,8,9*^*, Yahaya A. Yabo* ^1,2,3,5*^

*^1^Department of Neurosurgery, Medical Center - University of Freiburg, Freiburg, Germany*

*^2^Faculty of Medicine, University of Freiburg, Germany*

*^3^Microenvironment and Immunology Research Laboratory, Medical Center - University of Freiburg, Freiburg, Germany*

*^4^Department of Neurology, Clinical Neuroscience Center, University Hospital Zurich and University of Zurich, Zurich, Switzerland*

*^5^Department of Neurosurgery, University of Hospital Erlangen, Friedrich-Alexander-Universität Erlangen-Nürnberg, Germany*

*^6^Translational NeuroOncology Research Group, Medical Center - University of Freiburg, Freiburg, Germany*

*^7^Center for NeuroModulation (NeuroModul), University of Freiburg, Freiburg, Germany*

*^8^Department of Neurological Surgery, Northwestern University Feinberg School of Medicine, Chicago, USA*

*^9^German Cancer Consortium (DKTK), partner site Freiburg, Germany*

**Co-corresponding authors: Dieter Henrik Heiland**, E-mail: [dieter.henrik.heiland@uniklinik-freiburg.de](mailto:dieter.henrik.heiland@uniklinik-freiburg.de)**, Yahaya Abubakar Yabo:** E-mail: [yahaya.yabo@uk-erlangen.de](mailto:yahaya.yabo@uk-erlangen.de)

**
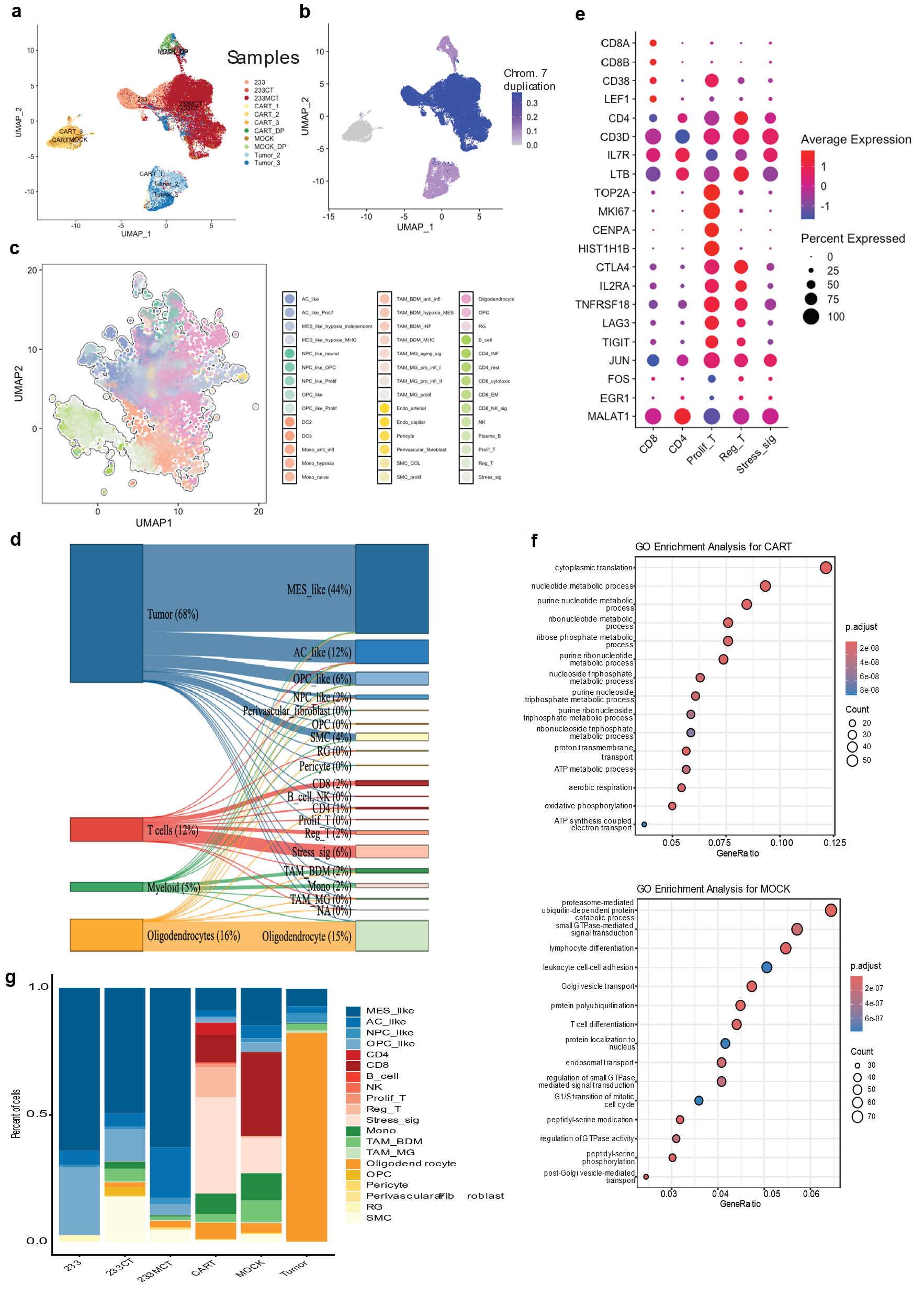
**

**Fig. S1: scRNA-seq data cell type annotation and gene ontology enrichment analysis.**

**(a)** UMAP representation showing individual samples and replicates in the merged dataset **(b)** Feature plot showing chromosome 7 duplication in tumor cells **(c)** UMAP showing cell types mapped to GBmap reference dataset **(d)** Sankey plot showing the similarity between our cell type annotation and the predicted GBmap cell type **(e)** Dotplot of markers expressed the different T cell subpopulation **(f)** Dotplots showing the top enriched gene ontology (GO) terms in CART and Mock groups (logfc.threshold = 0.5, adjusted p- and q-value cutoffs < 0.05) **(g)** Stacked barplot showing cell types proportions per condition following single cell profiling.

Conditions = 233CT = BTSC233 cell line + CART cells, 233CT = BTSC233 cell line + CAR T cells, 233MCT = BTSC233 cell line + Mock + CAR T cells, MOCK_DP= Mock double positive , CART_DP = CART double positive, MES-like = mesenchymal-like, AC = astrocytic-like, NPC = neural progenitor cell-like, OPC = oligodendrocyte progenitor cell-like, NK= natural killer cell, Prolif_T = proliferating T cells, Reg_T = regulatory T cells, Stress_sig = T cells with stress signature, Mono= monocyte, TAM_BDM = bone marrow-derived tumor-associated macrophages, TAM_MG = microglia derived tumor-associated macrophages, RG = radial glia, SMC= smooth muscle cells.


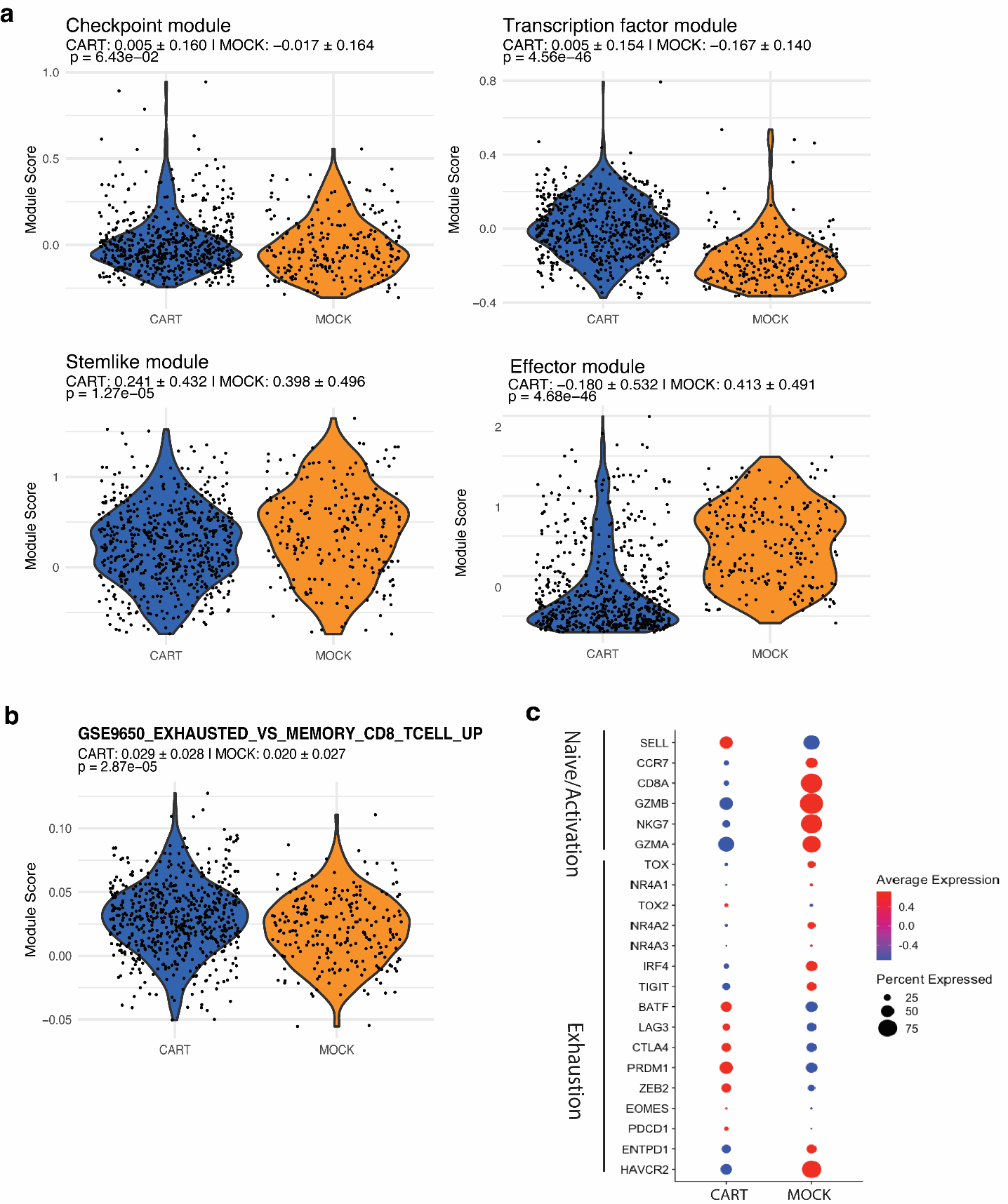


**Fig. S2:** **Program-level module scoring and marker expression of exhaustion-like features in CART and Mock T cell**

**(a)** Violin plots showing Seurat AddModuleScore values for four orthogonal gene programs across single cells in CART versus MOCK consisting of Checkpoint (inhibitory receptor module), Transcription factor Exhaustion (exhaustion-associated transcription factor module), Stemlike (progenitor/stem-like module), and Effector (cytotoxic/effector differentiation module). Mean ± SD and p-values are indicated on the plot. **(b)** Violin plot of an independent MSigDB reference signature (GSE9650_EXHAUSTED_VS_MEMORY_CD8_TCELL_UP) scored per cell using AddModuleScore. **(c)** Dot plot of selected activation/naïve and exhaustion/dysfunction-associated markers indicating the fraction of cells expressing each gene and color indicates scaled average expression.


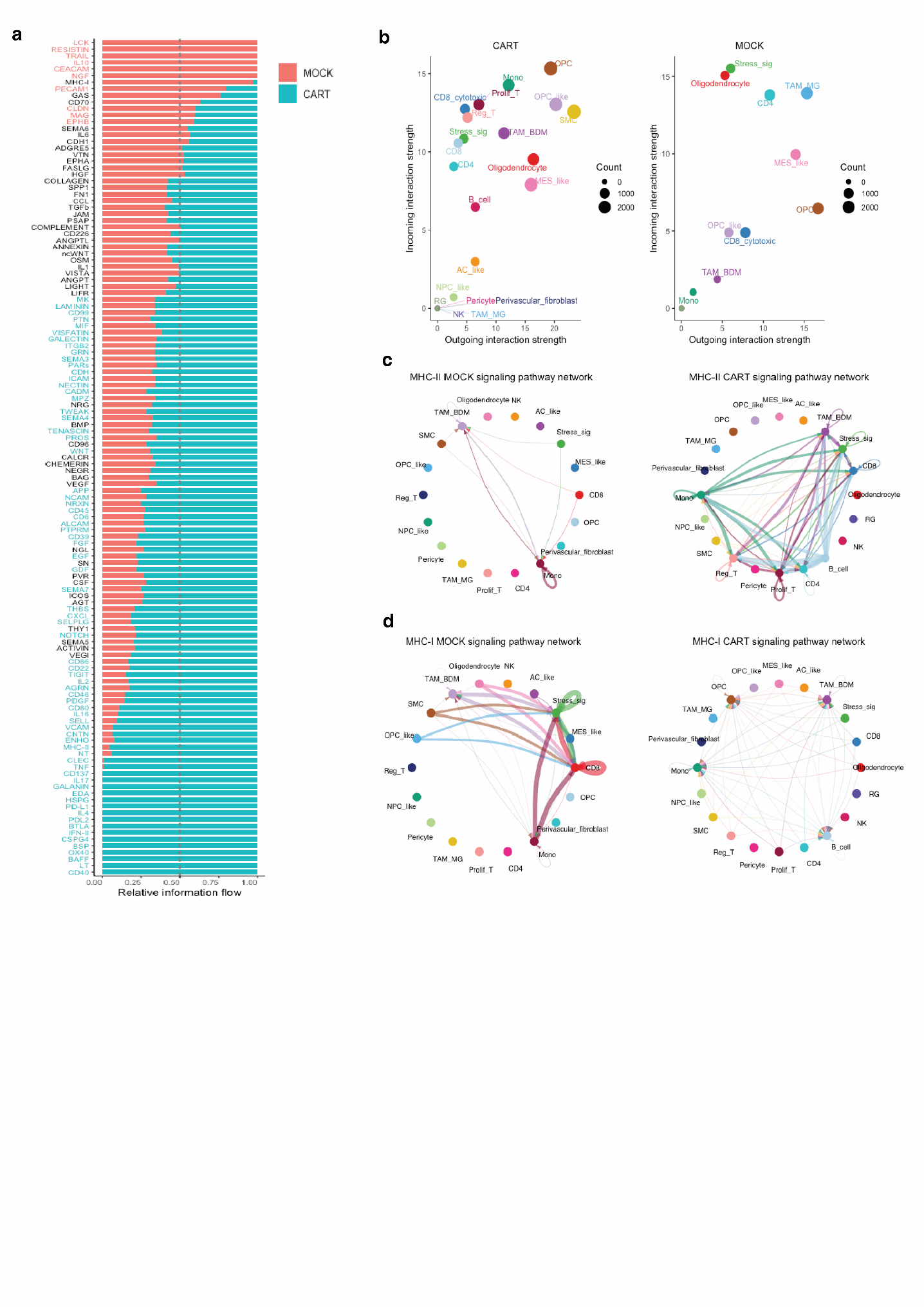


**Fig. S3: Cell to cell signaling analysis between CD8 T cells and other cells in the GB TME**

**(a)** Active signaling pathways in CD8 CAR-T and Mock group **(b)** Incoming and outgoing interactions between CD8 T cells and other cell types in Mock and CAR-T groups **(c and d)** Circle plots showing the signaling directions of MHC II and MHC I signaling pathways activated in the CAR-T cell and MOCK group.


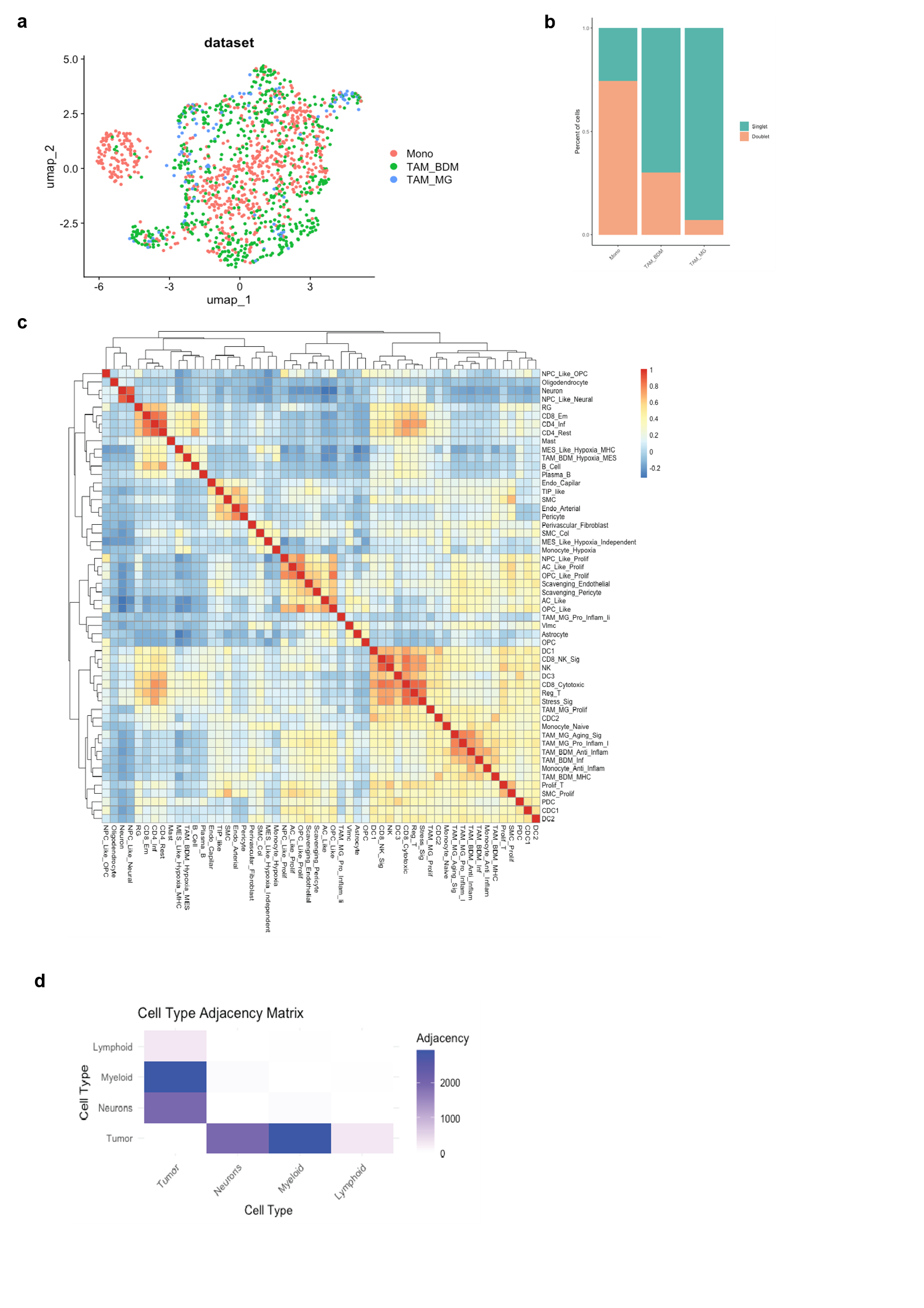


**Fig. S4: Spatial proximity of myeloid and tumor cells**

**(a)** UMAP showing the re-clustered myeloid cells with doublet scores annotated for different myeloid cell types **(b)** Stacked bar plots showing the distribution of cells with singlet and doublet scores across the different myeloid cell types **(c)** Correlation analysis of the proximity of cell types with each other in the spatial transcriptomics datasets **(d)** Heatmap of adjacency matrix scores showing a high proximity of myeloid and tumor cells in the spatial transcriptomics datasets.


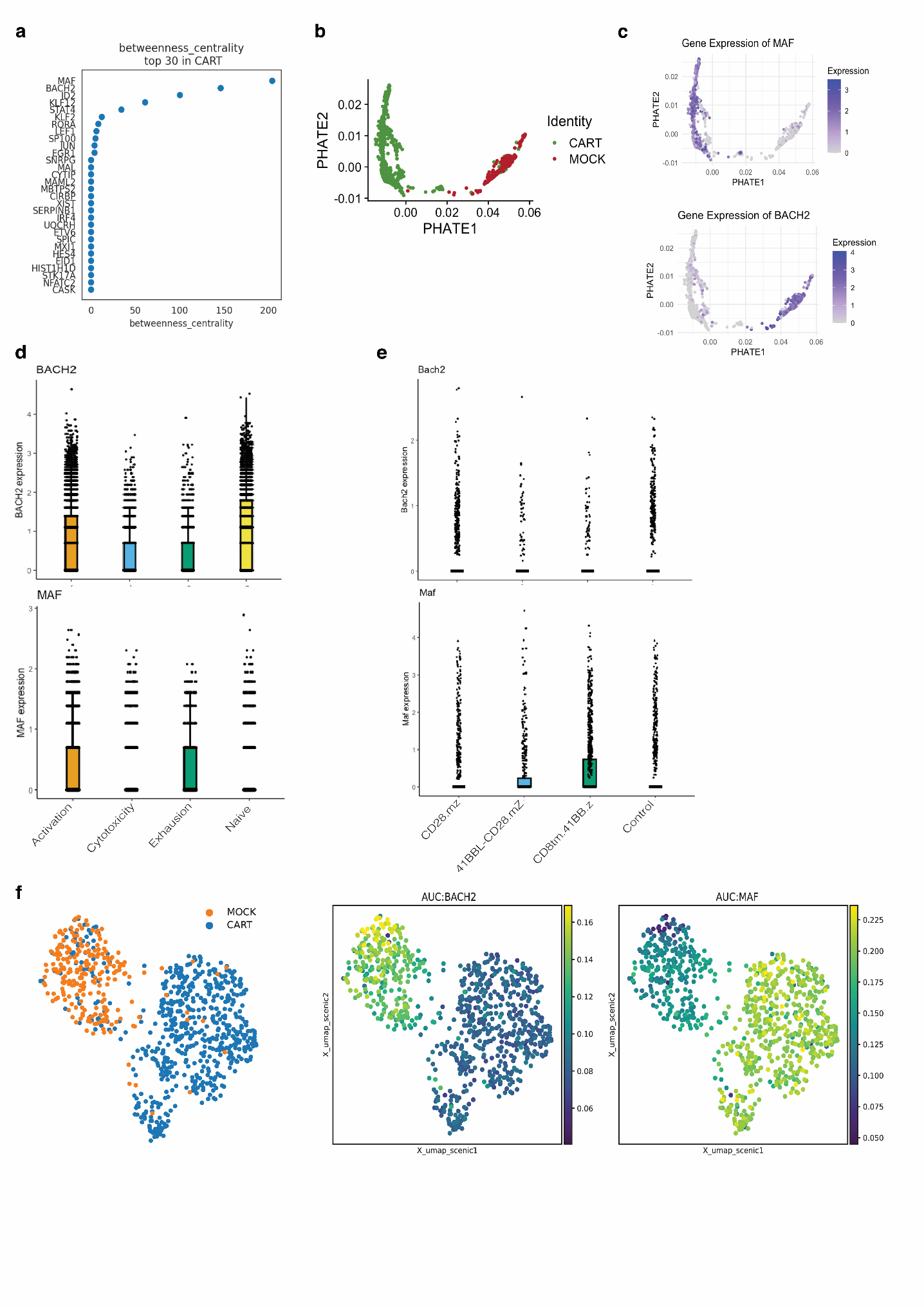


**Fig S5: Gene expression and transcription factor activity of MAF and BACH2**

**(a)** Top TFs ranked based on degree and betweenness centrality scores in the CD8 CAR-T group **(b)** Phate map showing CART and MocK groups differently clustered along the Phate trajectory **(c)** Feature plots of MAF and BACH2 on the Phate map showing alternate expression of corresponding to CART and MOCK groups respectively **(d)** Box plot showing the expression of BACH2 and MAF in CD8 T cells from human GB samples **(e)** Box plot showing the expression of BACH2 and MAF in CD8 T cells from different CAR constructs in mouse GB model **(f)** SCENIC analysis showing the activity of BACH2 and MAF.
